# Bioactive metabolites of *Streptomyces misakiensis* display broad-spectrum antimicrobial activity against multidrug-resistant bacteria and fungi

**DOI:** 10.3389/fcimb.2023.1162721

**Published:** 2023-04-24

**Authors:** Rewan Abdelaziz, Yasmine H. Tartor, Ahmed B. Barakat, Gamal EL-Didamony, Marwa M. Gado, Adina Berbecea, Habil dr. Isidora Radulov

**Affiliations:** ^1^ Department of Microbiology, Faculty of Science, Ain Shams University, Cairo, Egypt; ^2^ Department of Microbiology, Faculty of Veterinary Medicine, Zagazig University, Zagazig, Egypt; ^3^ Department of Botany and Microbiology, Faculty of Science, Zagazig University, Zagazig, Egypt; ^4^ Department of Soil Science, University of Life Science”King Mihai I” from, Timioara, Romania

**Keywords:** multidrug-resistant pathogens, antimicrobial biomaterials, antibacterial activity, antifungal activity, *streptomyces* metabolites

## Abstract

**Background:**

Antimicrobial resistance is a serious threat to public health globally. It is a slower-moving pandemic than COVID-19, so we are fast running out of treatment options.

**Purpose:**

Thus, this study was designed to search for an alternative biomaterial with broad-spectrum activity for the treatment of multidrug-resistant (MDR) bacterial and fungal pathogen-related infections.

**Methods:**

We isolated *Streptomyces* species from soil samples and identified the most active strains with antimicrobial activity. The culture filtrates of active species were purified, and the bioactive metabolite extracts were identified by thin-layer chromatography (TLC), preparative high-performance liquid chromatography (HPLC), nuclear magnetic resonance (NMR) spectroscopy, and gas chromatography-mass spectrometry (GC-MS). The minimum inhibitory concentrations (MICs) of the bioactive metabolites against MDR bacteria and fungi were determined using the broth microdilution method.

**Results:**

Preliminary screening revealed that *Streptomyces misakiensis* and *S. coeruleorubidus* exhibited antimicrobial potential. The MIC_50_ and MIC_90_ of S. misakiensis antibacterial bioactive metabolite (ursolic acid methyl ester) and antifungal metabolite (tetradecamethylcycloheptasiloxane) against all tested bacteria and fungi were 0.5 μg/ml and 1 μg/mL, respectively, versus *S. coeruleorubidus* metabolites: thiocarbamic acid, *N,N*-dimethyl, S-1,3-diphenyl-2-butenyl ester against bacteria (MIC_50_: 2 μg/ml and MIC_90_: 4 μg/mL) and fungi (MIC_50_: 4 μg/ml and MIC_90_: 8 μg/mL). Ursolic acid methyl ester was active against ciprofloxacin-resistant strains of *Streptococcus pyogenes*, *S. agalactiae*, *Escherichia coli, Klebsiella pneumoniae*, and *Salmonella* enterica serovars, colistin-resistant *Aeromonas hydrophila* and *K. pneumoniae*, and vancomycin-resistant *Staphylococcus aureus*. Tetradecamethylcycloheptasiloxane was active against azole- and amphotericin B-resistant *Candida albicans*, *Cryptococcus neoformans*, *C. gattii, Aspergillus flavus, A. niger*, and *A. fumigatus*. Ursolic acid methyl ester was applied *in vivo* for treating *S. aureus* septicemia and *K. pneumoniae* pneumonia models in mice. In the septicemia model, the ursolic acid methyl ester-treated group had a significant 4.00 and 3.98 log CFU/g decrease (*P* < 0.05) in liver and spleen tissue compared to the infected, untreated control group. Lung tissue in the pneumonia model showed a 2.20 log CFU/g significant decrease in the ursolic acid methyl ester-treated group in comparison to the control group. The haematological and biochemical markers in the ursolic acid methyl ester-treated group did not change in a statistically significant way. Moreover, no abnormalities were found in the histopathology of the liver, kidneys, lungs, and spleen of ursolic acid methyl ester-treated mice in comparison with the control group.

**Conclusion:**

*S. misakiensis* metabolite extracts are broad-spectrum antimicrobial biomaterials that can be further investigated for the potential against MDR pathogen infections. Hence, it opens up new horizons for exploring alternative drugs for current and reemerging diseases.

## Introduction

Antimicrobial resistance (AMR) develops when bacteria and fungi lose their ability to respond to antimicrobials, making infections more difficult to treat and consequently having greater rates of morbidity and mortality. AMR has been a major concern because of the dramatic increase and huge challenge to the effective treatment of serious infections that are resistant to available antibiotics (Economou and Gousia, 2015). Additionally, the overuse/abuse of antibiotics in humans and food animals has resulted in the global dissemination of multidrug-resistant (MDR) bacteria and resistance genes, which are recently considered emerging contaminants for the environment (Abd El-Aziz et al., 2021; Ma et al., 2021; Saber et al., 2022; Xu et al., 2022). Moreover, the dissemination of MDR bacteria to human and animal food is facilitated by the application of animal manure in agriculture (Zalewska et al., 2021). This situation prompted researchers to develop new and more effective antimicrobial agents for combating AMR (Sugden et al., 2016). Due to the rising expense and difficulties in generating new antibiotics, they are shifting their attention to investigating alternative antibiotics with a reduced risk of developing AMR. Natural products are promising despite the high expectations placed on synthetic compounds with effective antimicrobial activities (Arasu et al., 2013). Several investigations have been conducted to find novel drugs from microbial origins (Charousová et al., 2017; Sawa et al., 2018; Kumar et al., 2020).

Actinobacteria are a Gram-positive, ubiquitously distributed filamentous bacterial phylum and contain one of the largest bacterial genera, *Streptomyces*, which is an important source for the production of various naturally derived antimicrobial agents (Barka et al., 2016). The bioactive metabolites of actinobacteria have antifungal, antibacterial, antiparasitic, antiviral, insecticidal, anticancer, antioxidant, and anti-inflammatory activities. These metabolites have a considerable impact on the control of many infectious diseases as well as the pharmaceutical industry’s development (Barka et al., 2016; Rajaram et al., 2020; Abdelaziz et al., 2022). A pentacyclic triterpenoid known as ursolic acid has been linked to a variety of biological activities (Gu et al., 2015). Although literature on thiocarbamic acid, *N,N*-dimethyl, S-1,3-diphenyl-2-butenyl ester has not yet been found, it is known that urea-based chemicals are carbarmic acid derivatives (Serban, 2019). Thiocarbamic acid, *N,N*-dimethyl, S-1,3-diphenyl-2-butenyl ester shares structural similarities with the widely prescribed anti-diabetic medication sulfonylurea (Ajilore et al., 2021). One of the metabolites contained in the extracts of *Nicotiana tabacum* leaves is tetradecamethyl cycloheptasilloxane that has antimicrobial potential (Bamigboye et al., 2021). Microorganisms that can produce novel compounds or useful templates for the production of new antibiotics are being discovered as a result of the screening of novel strains that have not yet been tested for their antibacterial activity (Donadio et al., 2007). Since the environment can affect microbial metabolism, it is also recommended to search for the original sources of microorganisms. As a result, bacteria that produce antibiotics have been isolated from a variety of habitats, including the endophytes of terrestrial plants and sea organisms, among others (Arasu et al., 2013).

Since MDR can have a major negative impact on one’s health, either directly or through transferring characteristics to other pathogens, the discovery of innovative antimicrobial strategies is one of the biggest medical breakthroughs for ensuring the welfare and health of both humans and animals. Given the variety and complexity of microorganisms, the majority of these strategies require additional research to achieve more effective and satisfying results. As part of the World Health Organization’s efforts to address growing global AMR, the first list of MDR priority pathogens was published to guide and promote the development of new antibiotics. The list highlights the threat of MDR Gram-negative bacteria (including *Klebsiella* species, *Escherichia coli*, *Pseudomonas aeruginosa*, and *Salmonella* species) and *Staphylococcus aureus* (WHO, 2017). The opportunistic yeast *Candida albicans* can cause infections ranging from superficial mucosal to fatal systemic candidiasis (Tartor et al., 2022). In immunocompromised patients, cryptococcosis is a serious opportunistic infection that frequently results in pulmonary infection and meningitis. Amphotericin B, fluconazole, and 5-flucytosine are the available antifungal treatment. The treatment is hampered by antifungals’ high toxicity, rising rate of resistance, and inability of the antifungals to cross the blood–brain barrier (Spadari et al., 2020). *Aspergillus* species have recently caused an increase in the number of life-threatening acute invasive infections due to an increase in the number of patients undergoing chemotherapy, bone marrow or solid organ transplantation, and intensive corticosteroid therapy. Also, in patients with severe COVID-19 receiving immunosuppressive therapy, pulmonary aspergillosis must be regarded as a serious and potentially fatal consequence (Machado et al., 2021). So it is urgent to make continuous efforts toward efficient treatments.

Taken together, the present study was designed for the isolation and identification of *Streptomyces* species from soil samples, purification, characterization, and testing the antimicrobial potential of the bioactive secondary metabolites against a wide range of MDR bacterial and fungal pathogens. Moreover, for the evaluation of the therapeutic activity of the effective antimicrobial biomaterial, an *in vivo* study was performed.

## Material and methods

### Isolation and identification of *Streptomyces* species

A total of one hundred rhizosphere clay soil samples were collected from Sharkia Governorate, Egypt. The samples were spread onto starch-nitrate agar medium supplemented with 50 μg/ml of cyclohexamide and nystatin (Oxoid, Cambridge, UK) and incubated at 37°C for 5 days (Bae et al., 1972). After incubation, a grey colony that secretes red pigment was selected and purified by repeated streaking onto starch-nitrate agar medium. The recovered isolates were identified using the International *Streptomyces* Project’s descriptions (ISP) of mycelium shape, color, substrate mycelium, melanin production, and the soluble pigment (Shirling and Gottlieb, 1966). The analysis of the sugar components in the whole-cell hydrolysate and the isomer of diaminopimelic acid (DAP) within the cell membrane was performed as previously described (Marmur, 1961). The micromorphological characteristics of the isolates grown on starch-nitrate agar at 28°C for 14 days were observed using a bright-field light microscope (Nikon, China) and a scanning electron microscope (Joel, JSM-6360LA, Japan).

The active isolates were confirmed molecularly by PCR amplification of 16S rRNA gene using forward primer (5′-AGAGTTTGATCMTGGCTCAG-3′**)** and reverse primer (5′-TACGGYTACCTTGTTACGACTT-3′) (Lagacé et al., 2004). The amplicons were size-confirmed by electrophoresis using 1.2% agarose gel (Applichem, Germany, GmbH) stained with 0.5 μg/ml ethidium bromide (Sigma-Aldrich, MO, USA), and the identities were further verified by DNA sequencing in the forward and reverse directions on an Applied Biosystems 3130 automated DNA sequencer (ABI, 3130, USA) using a ready-reaction Bigdye Terminator V3.1 cycle sequencing kit (Perkin-Elmer/Applied Biosystems, Foster City, CA; Cat. No. 4336817). Using the Basic Local Alignment Search Tool (BLAST) on the National Center for Biotechnology Information (NCBI) website, the DNA sequences were compared to the GenBank sequences (www.ncbi.nlm.nih.gov). The MEGA11 (www.megasoftware.net) was used to analyze nucleotide sequences.

### Bacterial and fungal isolates

A total of 143 non-duplicate isolates including 14 *Staphylococcus aureus*, 8 *Listeria monocytogenes*, *Bacillus cereus* ATCC 36621, 12 *Streptococcus equi*, 4 *Streptococcus pyogenes*, 7 *Streptococcus agalactiae*, 17 *Escherichia coli*, 2 *Pseudomonas aeruginosa*, 13 *Salmonella enterica*, 11 *Klebsiella pneumoniae*, 17 *Aeromonas hydrophila*, and 1 *Flavobacterium columnare*, 19 *Candida albicans*, 3 *Cryptococcus neoformans*, 3 C*. gattii*, 3 *Aspergillus flavus*, 7* A. niger*, and 1 A*. fumigatus* were involved in this study. The source of each isolate is listed in **Table 1S**. All isolates were identified using phenotypic and molecular methods using genus- and species-specific primers (**Table 2S**) and kept at −20°C for subsequent use in brain heart infusion broth or Sabouraud dextrose broth (Oxoid, USA) containing 20% glycerol. Serotyping of *Salmonella* isolates was carried out using commercially available antisera (Denka Seiken Co., Ltd., United Kingdom) in accordance with the antigenic profile (Kauffmann, 1957).

### Antimicrobial susceptibility testing

The susceptibility of 143 isolates to the commonly used antimicrobials was examined using the disc diffusion method. Inhibition zone diameters for each antimicrobial determining a resistant, intermediate, or sensitive result were interpreted following the Clinical and Laboratory Standards Institute (CLSI) guidelines (CLSI, 2020). To confirm disc diffusion results, minimum inhibitory concentrations (MICs) were determined for colistin, ciprofloxacin, tigycycline, and vancomycin against Gram-negative bacteria, *Salmonella* isolates, and *S. aureus*, respectively, using the broth microdilution method (CLSI, 2020). CLSI recommended MICs breakpoint for ciprofloxacin, tigycycline, colistin, and vancomycin were used (≥ 1 µg/ml, >2 µg/ml, ≥ 4 µg/ml, and > 8 µg/ml, respectively) (CLSI, 2020).

### Screening for *Streptomyces* species antimicrobial activity

*Streptomyces* species isolates were evaluated for their antimicrobial activity toward the tested bacteria and fungi isolates using the agar plug method (Ekundayo et al., 2014). Briefly, an inoculum (1.5× 10^8^ CFU/mL) of fresh bacterial or fungal culture was inoculated on the surface of Muller-Hinton agar (MHA) plates or Sabouraud dextrose agar (Thermo Fisher Scientific Oxoid Ltd., Basingstoke, Hampshire, United Kingdom), and then *Streptomyces* colonies that had been cut with a sterile cork borer (8 mm) were deposited. The inoculated plate was incubated at 37°C for 24 h for bacteria and 28°C for 48 h for fungi. The results were recorded as “very strong” (zone diameter ≥ 31 mm), “strong” (21-30 mm), “moderate” (11-20 mm) or “weak” (10 mm) (Sahin and Uğur, 2003).

### Extraction of *Streptomyces* species metabolite extracts and testing their antimicrobial potential

A slant culture of each *Streptomyces* species was inoculated in 100 mL ISP-2 medium in a 200-mL Erlenmeyer flask and incubated at 30°C for 5 days on an orbital shaker at 200 rpm. The broth culture (100 mL) was centrifuged at 8000 rpm for 15 min, shaken vigorously on a flask shaker, and then filtered using filter paper (Whatman No. 1). Then ethyl acetate fractions of *S. misakiensis* metabolites and diethyl ether for *S. coeruleorubidus* metabolites were concentrated using a rotary vacuum evaporator (Micro Technologies, Myanmar, United States) at 40°C. The crude extracts were resuspended in 1 mL of 96% ethanol for washing and kept at −20°C (Mohamed et al., 2017).

The antimicrobial activity of extracellular crude metabolites was determined by an agar-well diffusion test (Chaudhary et al., 2013). The tested organism was adjusted to 1.5× 10^8^ CFU/mL and then inoculated on MHA plates. Wells were made using a sterile cork borer number 4 and 100 µL of each metabolite was added. Plates were incubated at 37°C for 24 h (for bacteria) or 28°C for 48 h (for fungi). The diameters of the inhibition zone were measured and interpreted in accordance with Sahin and Uğur (2003).

### Identification of bioactive metabolites

Thin-Layer Chromatography (TLC) (Sigma-Aldrish, Germany) was used to evaluate the extract initially on silica gel paper chromatography. To choose the solvent system capable of demonstrating greater resolution, TLC was conducted using various solvent systems with various polarities, such as chloroform: ethyl acetate (3:9) and toluene: ethyl acetate: formic acid (7:3:0.2) for both *Streptomyces* species metabolites. The aforementioned *Streptomyces* metabolite extracts were administered *via* capillary tubes to pre-coated TLC plates and then developed in a TLC chamber using the appropriate mobile phase. A 10 µL aliquot of the extract solutions was applied to the TLC paper. The produced TLC plates were air dried before being inspected in the UV TLC viewer under UV at both 254 nm and 366 nm. The rate of flow (Rf) values of the observed spots were calculated (Gaurav et al., 2020). Each band was scraped off individually, extracted with methanol, and put into a distinct vial. Then, an agar-well diffusion test was used to check each band’s antibacterial and antifungal activities. The preparative high-performance liquid chromatography (HPLC) (Good Science Instrument Technology Co., Ltd., Tianjin, China) was used to identify the active eluent compounds from TLC plates (Maleki and Mashinchian, 2011).

Gas chromatography-mass spectrometry (GC-MS) was used to examine the metabolites. It was done with a thermal mass spectrometer detector (ISQ Single Quadrupole Mass Spectrometer) and a TRACE GC Ultra Gas Chromatograph (THERMO Scientific Corp., USA). A TR-5 MS column (30 m x 0.32 mm i.d., 0.25 μm film thickness) was installed in the GC-MS system. The following temperature program was used for the analyses, which used helium as the carrier gas at a flow rate of 1.0 mL/min and a split ratio of 1:10: 60°C for 1 min, followed by a 4.0°C/min increase to 240°C and a 1 min hold. At 210°C, the injector and detector were maintained. One μL of the diluted samples (1:10 hexane, v/v) was injected. Using a spectral range of 40–450 m/z and electron ionization at 70 eV, mass spectra were produced. AMDIS software (www.amdis.net) was used to identify the chemical components of the metabolite, which were then determined by their retention indices (relative to n-alkanes C8-C22), mass spectra matching to standards (when available), Wiley spectral library collection, and National Institute of Standards and Technology (NIST) library database (Managamuri et al., 2017). The obtained data were verified using IR and NMR.

An IR spectrophotometer (ThermoFisher Nicolete IR IS10-USA) was used to scan the compounds’ infrared spectra at a range of 400 and 4000 cm^-1^ using ethyl acetate solution for *S. misakiensis* and diethyl ether for *S. coeruleorubidus* metabolites. Plots of the spectra’s intensity and wave number were made. Maximum and minimum resolutions, as well as the number of peaks, were measured in this spectrum range (Maleki and Mashinchian, 2011). Nuclear magnetic resonance (NMR) spectroscopy (Thermo Scientific Multiskan Sky High Microplate Spectrophotometer, Germany) is an analytical technique for obtaining detailed structural and quantitative information on metabolites. The data obtained were compared with those of the similar compounds produced by *S. misakiensis* and *S. coeruleorubidus* (Motohashi et al., 2008).

### Determination of the minimum inhibitory concentration of the identified metabolites

The broth microdilution method was performed using 96-well polystyrene microtitre plates (Costar, Corning Inc., USA) for the detection of MIC values of *Streptomyces* species metabolites against the tested bacterial and fungal isolates (Al-dhabi et al., 2020). Vacum dried ethyl acetate or diethyl ether metabolites of *Streptomyces* species were dissolved in tween 20 (1 gm/1 ml) then diluted (concentration range: 0.125 µg/ml to 512 µg/ml) in Mueller-Hinton broth in the case of bacterial isolates or in RPMI 1640 medium (Thermo Scientific™ Oxoid, Ltd., Basingstoke, Hampshire, United Kingdom) for testing fungal isolates. Subsequently, each well was inoculated with 100 µL of fresh bacterial culture suspension (5 × 10^5^) or fungal suspension (5 × 10^6^ CFU/ml) and the 96-well microtitre plate was incubated for 24–48 h at 37°C. Positive and negative controls were included. The lowest concentration that inhibits bacterial or fungal growth was established as the MIC.

### Efficacy of a bioactive metabolite for treating septicemia and pneumonia in mice

The effective secondary metabolite identified *in vitro* was tested *in vivo* in *S. aureus* and *K. pneumoniae* infection mouse models. A total of 112 five-week-old Albino mice (20–25g) were used for the septicemia and pneumonia infection models. They were kept in animal rooms at 25°C and allowed to acclimatize for 2 weeks prior to the start of the experiment to exclude any infection. The protocol of this experiment was approved by the Institutional Animal Care and Use Committee at Ain-Shams University (approval number ASU-SCI/MICR/2023/1/4).

For calculation of the 50% effective dose (ED50), five doses of the metabolite extract (100 mg/kg of body weight used as the highest dose) were created by using sequential 1.414-fold to 1.732-fold dilutions. Immediately after infection with *S. aureus* or *K. pneumoniae*, metabolite extract was injected into the tail veins of mice in the two groups. The survival rate on day seven following infection was used to calculate ED50s and 95% confidence intervals (Otani et al., 2003).

### *S. misakiensis* metabolites extract in a septicemia model

Mice were divided into seven groups (7 animals/group). Animals in groups G1, G2, and G3 were injected intraperitoneally (I.P.) with 0.2 ml of *S. aureus* suspension (1.5×10^8^) in phosphate buffered saline (PBS) (Otani et al., 2003). The first group (G1) was the control positive, non-treated group; G2 was infected and treated with 20 mg/kg of body weight gentamicin (GEN, Fulford, India, Ltd.) *via* the tail vein for seven days (Fierer et al., 1990); and G3 received 10 mg/kg of body weight metabolite extract dissolved in tween-20 (Sigma Aldrich, St. Louis, MO, USA) through the tail vein immediately after infection for seven days.

G4, G5, G6, and G7 were the negative control groups that received 0.2 ml of saline, tween-20 intraperitoneally, 10 mg/kg metabolite extract, and 20 mg/kg GEN, respectively for seven days. At day 7 postinfection, mice were sacrificed, and the liver and spleen were harvested for analysis of the bacterial burden. Aliquots of each organ homogenate in PBS were serially diluted, plated onto Mannitol Salt Agar and Baird-Parker Agar media (Oxoid, Cambridge, UK), incubated overnight at 37°C, and observed for viable colonies.

### *S. misakiensis* metabolites extract in the pneumonia model

The experiment protocol of Rodgers et al. (2019) was followed, which involved giving mice 50 µl of *K. pneumoniae* culture suspension in PBS (1.5×10^8^) intranasally, resulting in dissemination 24 hours after infection. The first group (G1) was the control positive group, G2 received 7.5 mg/kg GEN I.M. *via* the hind thigh muscle, and G3 received metabolite extract twice on the second day postinfection and then once a day for the next two days. The control negative groups (G4-G7) received 0.2 ml saline, tween-20 I.P., metabolite extract (7.5 mg/kg, I.M.), and GEN [7.5 mg/kg, I.M. (Rodgers et al., 2019)]. The antibacterial efficacy of metabolite extract in comparison with GEN for treating pneumonia was evaluated after three days postinfection based on the clinical signs and mortality rate that were recorded daily for three days. The total bacterial count of *K. pneumoniae* was estimated by a ten-fold serial dilution of the lung samples of mice that received GEN, metabolite extract and control groups. The appropriate dilutions were inoculated on MacConkey’s and eosin methylene blue (EMB) (Oxoid, Cambridge, UK) agar plates and incubated for 24 hours at 37°C.

### Survival rate of animals in septicemia and pneumonia models

The survival rate (SR) of mice in various groups was determined using the following formula: SR = Total number of mice/Total number of mice mortalities ×100 (Collins and Kays, 2014).

### Blood chemistry parameters

Blood samples were obtained from mouse groups in the septicemia model at day 7 postinfection and from the pneumonia model at day 3 postinfection for the determination of clinical chemistry parameters. Firstly, the mice were anaesthetized with 5 mg xylazine and 100 mg ketamine (Sigma Aldrich, St. Louis, MO, USA) (I.M.) (Parasuraman et al., 2010). Approximately 2 ml of blood were drawn from each mouse *via* the caudal vertebral vein with a 22 mm needle and immediately transferred into sterile tubes (Johnson & Johnson, Ramsey, MN, USA), which were allowed to coagulate and then centrifuged at 3500 rpm for 5 min to obtain serum. The serum total protein (TP), aspartate aminotransferase (AST), Alanine transaminase (ALT), urea, and creatinine levels in different animal groups were determined using Spinreact kits (Esteve De Bas, Girona, Spain) at a wavelength of 540 nm, in accordance with the methods of Kamimoto et al. (1985). Additionally, the consequences of renal damage such as urea and creatinine were measured using Spinreact kits (Esteve De Bas, Girona, Spain) in accordance with the methods established by Fossati and Prencipe (2010) and Murray (1984), respectively, by an automatic biochemical analyzer (Hitachi 902, Roche Diagnostics).

### Histopathological examination

The collected specimens from the liver and spleen from different groups in the septicemia model and the lungs from the pneumonia model were fixed in 10% neutral buffered formalin, dehydrated in ascending grades of alcohol, cleared in xylene, and embedded in melted paraffin wax. Paraffin 5-µm sections were obtained using a microtome (Thermo Scientific, Massachusetts, USA) and stained with hematoxylin and eosin (Suvarna et al., 2018).

### Data analysis

All the experiments were done in triplicate, and the results were displayed as means ± standard error (SE). A Shapiro-Wilk test was conducted in order to check for normality as described by Razali and Wah (2011). The data were analyzed using one-way analysis of variance (SAS Institute Inc, 2012). The differences between the survival rates of the studied groups were examined using the Chi-Square test (χ2) (SAS Institute Inc, 2012). Statistical significance was set at a *P*-value less than 0.05.

## Results

### Characteristic features of *Streptomyces* species isolates

Among the 58 Streptomyces isolates screened, two isolates displayed antimicrobial activity. *Streptomyces* species isolates grew well on some media but only moderately or barely on others. *Streptomyces misakiensis* grew well and produced gray-white aerial mycelium on starch-nitrate agar medium, but the substrate mycelium was red and did not produce soluble pigment. *S. misakiensis* grew moderately on yeast malt agar medium (ISP-2) and produced white-gray aerial mycelium but no substrate-soluble pigment. On glycerol asparagine agar medium (ISP-5), *S. misakiensis* grew slowly with grey aerial mycelium. On tyrosine agar medium (ISP-7), *S. misakiensis* gave weak growth, yellowish-gray, white aerial mycelium, but the substrate mycelium was dark yellow without soluble pigments. On medium (ISP-6), the isolate gave moderate growth and pale gray aerial mycelium, but the substrate mycelium was yellowish without soluble pigment. Melanoid pigmentation and soluble pigmentation were not seen. The colour of *S. coeruleorubidus* conidia was grey on all ISP media except ISP-6, which was nil. High sporulation rates were observed on ISP-2 medium; good sporulation was found on ISP-3 and 4; and low sporulation was found on ISP-7.

The ability of *S. misakiensis* isolate to utilize various carbon compounds as the source of energy was indicated by heavy growth on ISP media supplemented separately by the following carbon sources: glucose, arabinose, xylose, inositol, mannitol, fructose, rhamnose, sucrose, and raffinose. *S. coeruleorubidus* has the ability to grow on ISP media supplemented with all tested carbon sources. A 14-day-old *S. misakiensis* culture on starch-nitrate agar medium was examined microscopically to exhibit short filamentous mycelia with few straight and smooth surface spore chains (**Figure S1A**). *S. coeruleorubidus* spore chains were spiral with a hook end and a spiny surface (**Figure S1B**).

DNA sequencing of 1485-bp amplicons of the 16S rDNA gene (**Figure S2** **)** confirmed the identification of *S. misakiensis* and *S. coeruleorubidus* (GenBank accession numbers OP168477 and OP168352, respectively).

### Antimicrobial activity of *S. misakiensis* and *S. coeruleorubidus*

Preliminary antimicrobial activity screening of both *Streptomyces* species revealed that *S. misakiensis* has strong antimicrobial activity against Gram-positive bacteria (*S. aureus*, *L. monocytogenes*, *B. cereus*, *S. equi*, *S. pyogenes*, and *S. agalactiae*) as well as Gram-negative bacteria (*E. coli*, *Salmonella* species, *K. pneumoniae*, *F. columnare*, *A. hydrophila*, and *P. aeruginosa*). However, *S. coeruleorubidus* showed strong to moderate antimicrobial activity on *S. agalactiae*, *S. pyogenes*, *L. monocytogenes*, *B. cereus*, *A. hydrophila*, *E. coli*, *K. pneumoniae*, and *Salmonella* enterica, but didn’t inhibit *P. aeruginosa* and *F. columnare*. Furthermore, *S. misakiensis* has strong antifungal activity against *A. flavus*, *A. niger*, *C. neoformans*, *C. gattii*, and *C. albicans*, whereas *S. coeruleorubidus* has moderate to strong antifungal activity (**Table 1** and **Table 1S**). The secondary metabolites of both species were purified and identified for further application in an *in vivo* model.

**Table 1:** Antimicrobial activity of bioactive metabolites of *S*. *misakiensis* and *S. coeruleorubidus*.

Species (No. of strain)	Ursolic acid methyl ester			Thiocarbamic acid, *N,N*- dimethyl, S-1,3-diphenyl-2-butenyl ester		
	MIC Range (μg/mL)	MIC_50_ (μg/mL)	MIC_90_ (μg/mL)	MIC Range (μg/mL)	MIC_50_ (μg/mL)	MIC_90_ (μg/mL)
*S. aureus* (14)	0.125-2.0	0.5	2.0	0.00	–	–
*B. cereus* ATCC11778	0.5	NE	NE	0.00	–	–
*L. monocytogenes* (8)	0.125-1.0	NE	NE	4.0-8.0	NE	NE
*S. equi* (12)	0.125-1.0	0.5	1.0	0.00	–	–
*S. pyogenes* (4)	0.125-2.0	NE	NE	1.0-4.0	NE	NE
*S. agalactiae* (7)	0.125-1.0	NE	NE	1.0	NE	NE
*E. coli* (17)	0.125-1	0.5	1.0	0.5-4.0	2.0	4.0
*P. aeruginosa* (2)	0.5-1.0	NE	NE	0.00	–	–
*K. pneumoniae* (11)	0.125-1.0	0.25	0.5	1.0-4.0	2.0	2.0
*A. hydrophila* (17)	0.125-0.5	0.25	0.5	0.5-4.0	1.0	4.0
*Salmonella* species (13)	0.125-1.0	0.5	1.0	1.0-8.0	4.0	8.0
*F. columnare* (1)	0.5	NE	NE	0.00	–	–
Fungi	Tetradecamethylcycloheptasiloxane			Thiocarbamic acid, *N,N*-dimethyl, S-1,3-diphenyl-2-butenyl ester		
*C. albicans* (19)	0.125-2.0	0.5	2.0	1.0-8.0	4.0	8.0
*C. neoformans and C. gattii * (6)	0.125-1.0	NE	NE	0.00	–	–
*A. niger* (7)	0.125-1.0	NE	NE	2.0-16	NE	NE
*A. flavus* (3)	0.125-1.0	NE	NE	1.0	NE	NE
*A. fumigatus* (1)	0.5	NE	NE	0.00	–	–

NE: MIC50 and MIC90 were not estimated for species with less than 10 isolates.

### Identification of *Streptomyces* species metabolite extracts

TLC analysis of *Streptomyces* metabolite extract revealed six fractions. The two fractions, R2 and R3, showed antifungal and antibacterial activities, respectively, against the tested species. The Rf value of R2 was 4.5 cm and 3.5 cm for R3. The TLC results were confirmed by HPLC preparative, which gives six peaks as shown in **Figure S3**: 18.9, 19.409, 20.228, 21.615, 24.727, 29.392, 32.16, 34.7, and 35.2.

The GC-MS analysis of the purified compound from TLC was performed. The chemicals were identified based on their peak area, molecular weight, and molecular formula. The amount of substance in the active band is exactly proportional to this area. Ursolic acid methyl ester (Urs-12-en-28-oic acid, 3-hydroxy-, methyl ester, (3á)) is a novel antibacterial metabolite identified from *S. misakiensis* metabolites extrac, whereas tetradecamethylcycloheptasiloxane is the identified antifungal compound, as shown in **Figures S4**, **S5**.

The IR spectrum of the ursolic acid methyl revealed functional group spectra at: 32982, 1748, 1377, 1243, 1051, 929, 847, 620, and 456.18 cm^-1^ for ( OH) stretching alcohol, ( C-H aliphatic), ( C═ O), (ester) and ( C═ C), respectively (**Figure S6**). As shown in **Figure S7**, the 1H NMR spectrum was at 3.502, indicating that this antibiotic is mostly ursolic acid methyl ester (Urs-12-en-28-oic acid, 3-hydroxy-, methyl ester, (3á)). As shown in **Figure S8**, the IR spectrum of tetradecamethylcycloheptasiloxane displayed functional group spectra at 2981, 2865, 1056, 1033, 1010, and 520 cm^-1^. The NMR spectrum was at 80.02, as revealed in **Figure S9**, indicating that this antibiotic is mostly tetradecamethylcycloheptasiloxane.

*S. coeruleorubidus* diethyl ether extract was separated on TLC plates into three fractions (A, B, and C). The one active fraction Rf value for the strain was 2.0 cm, which has antibacterial and antifungal activity against the tested bacteria and fungi. HPLC analysis gives five peaks at 19.409, 20.228, 21.615, 24.727, and 29.392 (**Figure S10** **)**.

The metabolites of *S. coeruleorubidus*’ diethyl ether extract were analysed using GC-MS, and the results revealed that the antibacterial and antifungal compound thiocarbamic acid, *N,N*-dimethyl, S-1,3-diphenyl-2-butenyl ester, predominated (**Figure S11**). **Figures S12**, **S13** depict the IR and NMR spectra of thiocarbamic acid, *N,N*-dimethyl, S-1,3-diphenyl-2-butenyl ester.

### MICs of ursolic acid methyl ester, tetradecamethylcycloheptasiloxane, and thiocarbamic acid, *N,N*-dimethyl, S-1,3-diphenyl-2-butenyl ester

Antibacterial and antifungal activities of the bioactive compounds in terms of MIC are shown in **Table 1**. Ursolic acid methyl ester exhibited antibacterial activity against a variety of species of Gram-positive and Gram-negative bacteria with MIC values ranging from 0.125 to 2 μg/ml, whereas thiocarbamic acid, *N,N*-dimethyl, S-1,3-diphenyl-2-butenyl ester showed antimicrobial activity against *E. coli*, *K. pneumoniae*, *A. hydrophila*, and *S. pyogenes* (MIC 0.5 - 4 μg/ml). Some strains of *Salmonella enterica* serovars and *L*. *monocytogens* were inhibited with thiocarbamic acid, *N,N*-dimethyl, S-1,3-diphenyl-2-butenyl ester (MIC values of 1-8 μg/ml and 4-8 μg/ml, respectively). *S. aureus*, *S. equi*, and *P. aeruginosa* were resistant to thiocarbamic acid, *N,N*-dimethyl, S-1,3-diphenyl-2-butenyl ester. Concerning antifungal potential of both *Streptomyces* species metabolites extracts, MICs ranged from 0.125 to 1 μg/ml for tetradecamethylcycloheptasiloxane and 1 to 16 μg/ml for thiocarbamic acid, *N,N*-dimethyl, S-1,3-diphenyl-2-butenyl ester. Tetradecamethylcycloheptasiloxane was more effective on *C. albicans* than thiocarbamic acid, *N,N*-dimethyl, S-1,3-diphenyl-2-butenyl ester (MIC 0.125-2 μg/ml *vs* 1-8 μg/ml). Moreover, tetradecamethylcycloheptasiloxane was highly effective on *C. neoformans* and *C. gattii* but thiocarbamic acid, *N,N*-dimethyl, S-1,3-diphenyl-2-butenyl ester was not effective on both *Cryptococcus* species. The MIC_50_ and MIC_90_ of the antibacterial ursolic acid methyl ester and the antifungal tetradecamethylcycloheptasiloxane against all tested bacteria and fungi were 0.5 μg/ml and 1 μg/mL, respectively, versus thiocarbamic acid, *N,N*-dimethyl, S-1,3-diphenyl-2-butenyl ester against bacteria (MIC_50_: 2 μg/ml and MIC_90_: 4 μg/mL) and fungi (MIC_50_: 4 μg/ml and MIC_90_: 8 μg/mL).

### Efficacy of ursolic acid methyl ester for treating septicemia and pneumonia in mice

**Figure 1:**
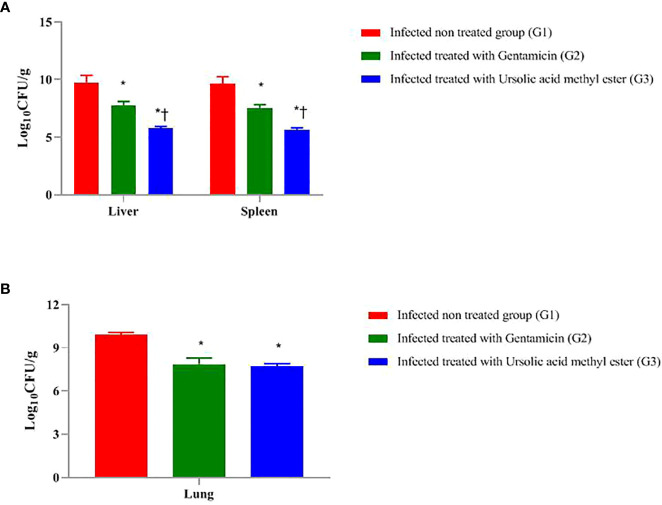
Total colony count (Log _10_ CFU g ^-1^) in liver and spleen of mice infected intraperitoneal with *S. aureus* **(A)** and the lungs of mice infected via the intranasal route with *K*. *pneumoniae* **(B)** and treated with gentamicin (10 mg/kg) and/or ursolic acid methyl ester (7.5 mg/kg). *significant difference with infected non treated group and † with gentamicin treated group (*P* < 0.05).

As presented in **Figure 1A**, at day 7 postinfection, the total bacterial burden in the liver sample of infected, non-treated G1 was 9.75 log CFU/g, followed by GEN-treated G2 (7.76 log CFU/g). The lowest bacterial count recorded was 5.75 log CFU/g in ursolic acid methyl ester**–**treated G3.

The highest total bacterial count in the spleen sample was 9.65 log CFU/g in G1, followed by G2 (7.54 log CFU/g). Ursolic acid methyl ester**-**treated G3 has the lowest bacterial count (5.67 log CFU/g). There were significant differences between G1 and G2, G2 and G3, and between G1and G3 (*P* < 0.05) (**Figure 1A** **)**.

There was a significant decrease in log CFU in liver tissue of GEN-treated mice compared to the infected, non-treated G1 (1.99 log CFU; *P* < 0.05). Similarly, there was a significant decrease in log CFU in the ursolic acid methyl ester**–**treated G3 compared to the control group (4.00 log CFU; *P* < 0.05). However, the group of mice treated with GEN was significantly higher in log CFU (2.01 log CFU; *P* < 0.05) than their counterparts treated with ursolic acid methyl ester (G3).

The GEN treated G2 had significantly lower log CFU (2.11 CFU; *P* < 0.05) in the spleen than the untreated group (G1). Furthermore, those treated with ursolic acid methyl ester had 3.98 fewer log CFU than the control group (*P* < 0.05). In comparison to the GEN-treated G2, the ursolic acid methyl ester-treated G3 reduced log CFU by 1.87 (*P* < 0.05; **Figure 1A**).

At day 3 postinfection, the microbial load in the lung sample in the pneumonia model, as shown in **Figure 1B**, was 9.88 CFU/ml in G1, 7.86 log CFU/ml in G2, and 7.68 Log CFU/ml in G3. There was a significant difference (*P* < 0.05) between G1, G2, and between G2, G3 and G1, G3.

In the septicaemia model, there were significant differences (*P* = 0.0324) between the survival rate in the examined groups, being 100% in ursolic acid methyl ester**-**treated G3, 80% in GEN-treated G2, and 20% in the infected, non-treated group G1. Regarding the pneumonia model, survival rates varied between 15% in G1, 70% in G2, and 100% in G3 (*P* = 0.0168).

### Liver and kidney functions of mice infected and treated with ursolic acid methyl ester and gentamicin

ALT and AST levels were high in both infected, non-treated G1 (52.87 ± 0.975; 63.33 ± 0.698 mg/dL) and GEN-treated G2 (84.37 ± 0.369; 92.17± 1.33 mg/dL) in the septicaemia model. Moreover, high liver enzyme levels were found in G1 (42.49 ± 0.606; 58.27 ± 0.433 mg/dL) and G2 (57.43 ± 1.131; 65.23 ± 1.068 mg/dL) in the pneumonia model. Ursolic acid methyl ester**-**treated G3 showed normal liver enzyme levels compared to GEN-treated G2 that showed high levels in septicaemia and pneumonia models, as listed in **Tables 3S**, **4S**. In the septicaemia and pneumonia models, all control negative groups, including tween-20 (G7), saline (G6), and ursolic acid methyl ester (G4), showed normal liver enzyme levels.

The total protein and albumin levels were low in G1 and GEN-treated G2 compared with the control negative groups. The total bilirubin and direct bilirubin were high in G1 and GEN-treated G2, followed by GEN-control negative G5. There were significant (*P* < 0.05) differences between all groups and the ursolic acid methyl ester-control negative G4 (**Tables 3S**, **4S**).

In the septicemia model, the highest levels in urea and creatinine were found in GEN-treated G2 (61.90 ± 0.606; 2.30 ± 0.254 mg/dL), infected, non-treated G1 (58.50 ± 0.952; 1.87 ± 0.086 mg/dL), and GEN-control negative G5 (58.44 ± 0.594; 1.90 ± 0.054 mg/dL). Ursolic acid methyl ester G3 showed normal urea and creatinine levels (40.33 ± 0.282; 1.13 ± 0.077 mg/dL) versus GEN-treated G2 that showed high levels.

Regarding animals in the pneumonia model, GEN-treated G2, GEN-control negative G5, and G1 recorded the highest urea and creatinine levels (**Tables 3S**, **4S**). Although animals in ursolic acid methyl ester**-**treated G3 (38.87 ± 0.588; 0.80 ± 0.057 mg/dL) have normal urea and creatinine levels, GEN-treated G2 displayed higher levels (58.13 ± 1.062; 1.93 ± 0.069 mg/dL). Normal levels of both urea and creatinine were detected in the control negative groups (G4, G6, and G7) of the septicemia and pneumonia models (**Tables 3S**, **4S**).

### Histopathological findings in different animal groups

**Figure 2:**
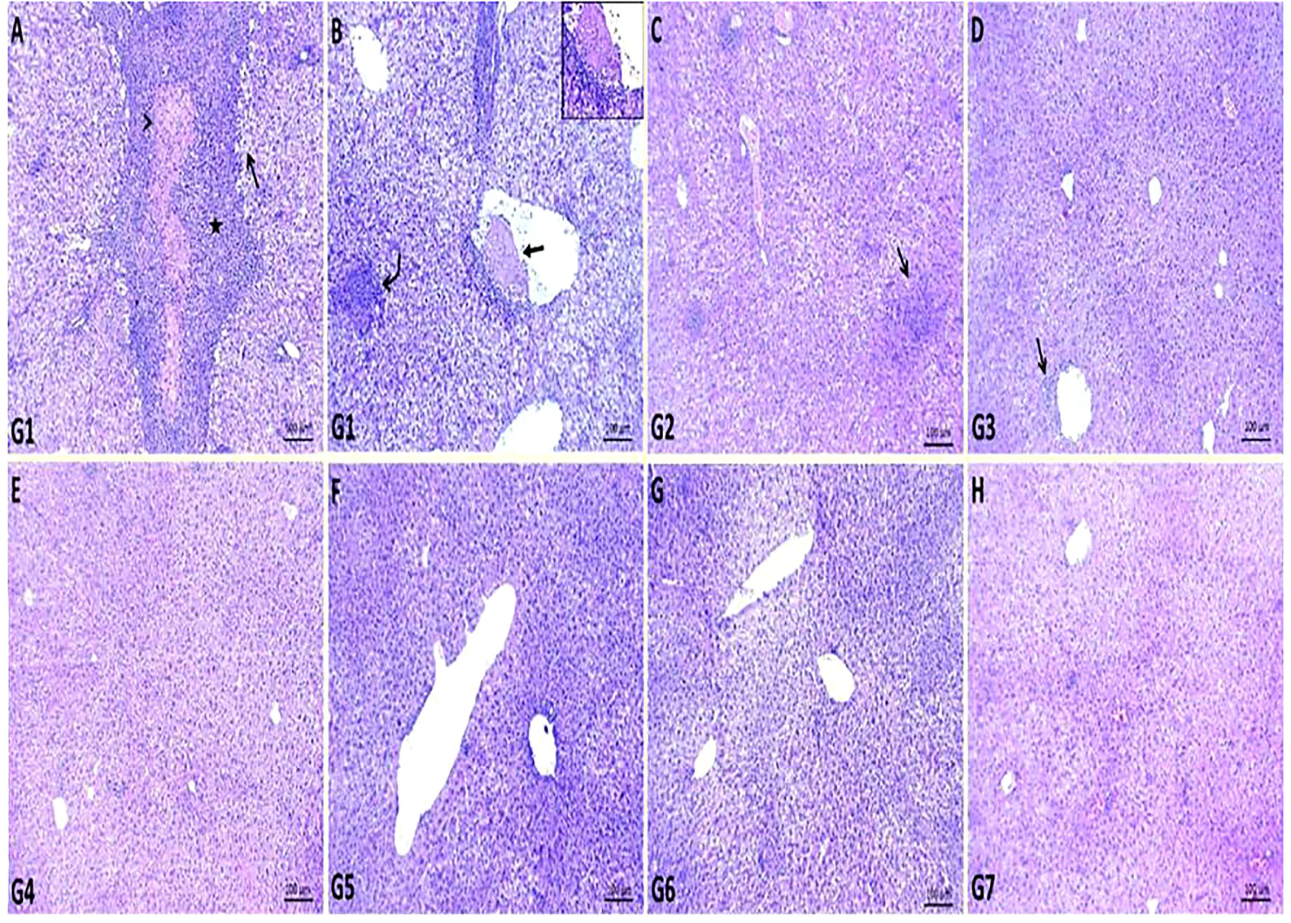
Representative photomicrographs of liver sections of different groups in *S. aureus* mouse model. **(A, B)**: areas of coagulative necrosis (arrowhead) surrounded by intense inflammatory cells infiltrates (star), focal area of microabscess (curved arrow), areas of steatosis (open arrow) and presence of attached thrombus within some hepatic blood vessel (closed arrow) in control positive group (G1), **(C)**: replacement of focal areas of hepatic parenchyma by leukocytic infiltrates (arrow) in gentamicin-treated group (G2), **(D)**: presence of few perivascular inflammatory cells infiltrations (arrow) in ursolic acid methyl ester*-*treated group (G3), **(E)**: normal histology of hepatic parenchyma and blood vessels in saline control negative group (G4), **(F)**: normal histomorphological structures of hepatic cords and central veins in tween-20 control negative group (G5), **(G)**: normal cytoarchitectures of hepatic cells and central veins in ursolic acid methyl ester control negative group (G6), **(H)**: relatively normal hepatocytes and hepatic vasculatures in gentamicin control negative group (G7). H&E, scale bar 100 μm.

Multiple areas of coagulative necrosis surrounded by intense inflammatory cell infiltrates and focal areas of microabscesses composed mainly of neutrophils were observed in G1. Most hepatic cells revealed degenerative changes such as hydropic degenerations and steatosis. Some hepatic blood vessels exhibited the presence of thrombus, which formed from a mass of fibrin threads and leukocytes attached within the tunica intima (**Figures 2A, B**). GEN-treated G2 showed restoration of most hepatic parenchyma with replacement of focal areas by leukocytic infiltrates (**Figure 2C** **)**, while the liver of ursolic acid methyl ester**-**treated G3 showed normal hepatic structures with the presence of a few perivascular inflammatory cell infiltrations (**Figure 2D** **)**. The liver of control negative groups (G4, G5, and G6) showed normal histomorphological structures of hepatic cords, central veins, and portal areas (**Figures 2E–H**).

**Figure 3:**
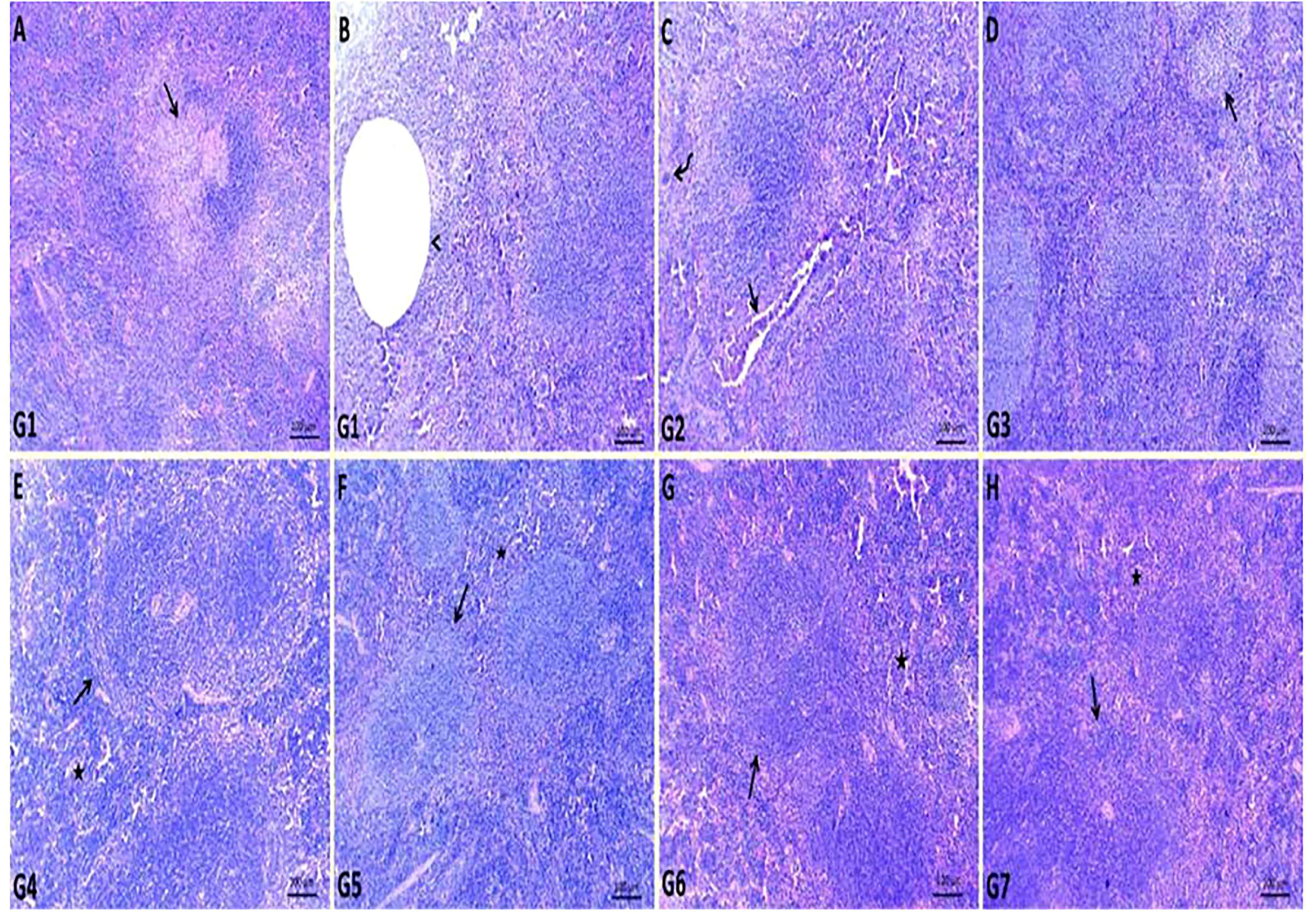
Representative photomicrographs of spleen sections of different groups in *S. aureus* mouse model. **(A, B)**: replaced number of white pulp either by necrotic materials (arrow) or cystic formation (arrow head) surrounded by necrotic debris in control positive group (G1), **(C)**: relatively normal white pulp with presence of large number of megakaryocytes (curved arrow) within red pulp and heavy infiltrated neutrophil, lymphocyte, macrophage within dilated splenic sinusoids and splenic blood vessels (arrow) in gentamicin treated group (G2), **(D)**: normal white and red pulp with presence of some mildly depleted white pulp (arrow) in ursolic acid methyl ester-treated group (G3), **(E)**: normal splenic white (arrow) and red pulp (star) in saline control negative group (G4), **(F)**: normal histomorphology of white pulp lymphoid zones (arrow) and red pulp (star) with splenic sinusoids in tween-20 control negative group (G5), **(G)**: mildly activated most of white pulp cells (arrow) and moderately infiltrated red pulp with hematopoietic elements (star) in ursolic acid methyl ester control negative group (G6), **(H)**: normal cytomorphology of germinal, mantle and marginal zones of white pulp (arrow) and infiltrated red pulp by large number of lymphocyte, neutrophils, and macrophages (star) in gentamicin control negative group (G7). H&E, scale bar 100 μm.

The spleen of G1 contained fewer white pulp cells due to eosinophilia and basophilic granular necrotic materials. Some white pulp lymphoid populations have been replaced by cystic formations surrounded by necrotic debris. Most examined sections contained a high number of megakaryocytes (**Figures 3A, B**). The spleen of GEN-treated G2 showed relatively normal white pulp with a large number of megakaryocytes within the red pulp and heavy neutrophil, lymphocyte, and macrophage infiltration within dilated splenic sinusoids and blood vessels (**Figure 3C**). In ursolic acid methyl ester-treated G3, there was apparent normal white and red pulp with some mildly depleted white pulp, as evidenced by a decreased number of lymphoid elements in both the germinal and mantle zones (**Figure 3D** **)**. Ursolic acid methyl ester-control negative G4 showed mildly activated white pulp cells and moderately infiltrated red pulp with hematopoietic elements (**Figure 3E** **)**. Control negative groups had normal histomorphology of white pulp lymphoid zones and red pulp with splenic sinusoids (**Figures 3F–H**).

**Figure 4:**
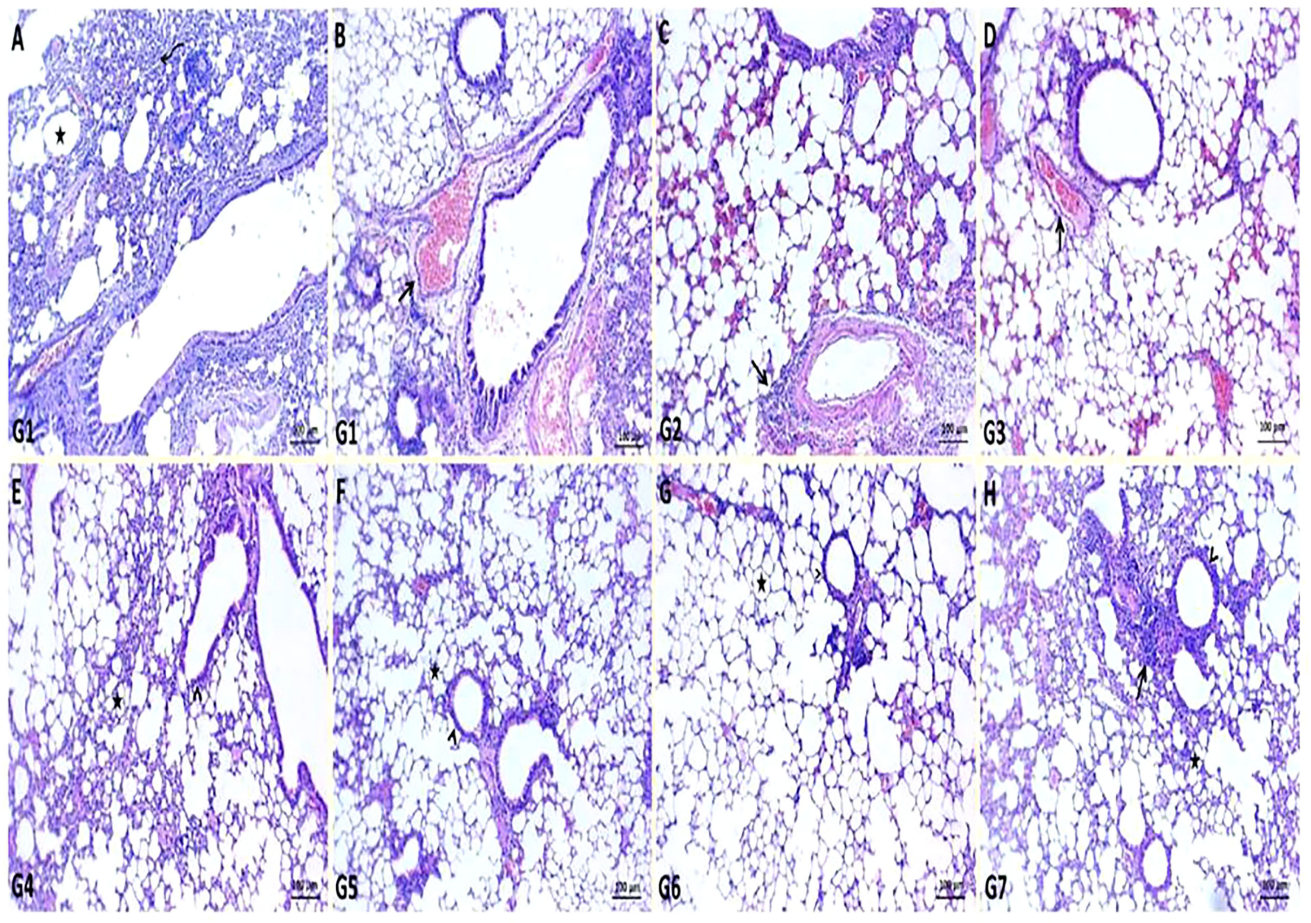
Representative photomicrographs of lung sections of different groups in *K*. *pneumoniae* mouse model. **(A, B)**: pneumonic changes (curved arrow) alternated with emphysematous changes (star) and dilated pulmonary blood vessels (arrow) in control positive group (G1), **(C)**: normal bronchiole (arrowhead) with prominent lymphoid follicles within their walls (arrow) and normal alveoli (star) in gentamicin treated group (G2), **(D)**: restoration of normal architectures of pulmonary tissue with perivascular inflammatory cell infiltrates (arrow) in ursolic acid methyl ester-treated group (G3), **(E)**: apparent normal bronchi and alveolar epithelium with presence some dilated vasculatures (arrowhead) in saline control negative group (G4), **(F)**: normal pulmonary tissue, bronchiole (arrowhead), and alveolar structures (star) in tween-20 control negative group (G5), **(G)**: normal bronchi, bronchioles (arrowhead), alveolar ducts, and alveoli (star) in ursolic acid methyl ester control negative group (G6), **(H)**: normal histomorphological structures of epithelial lining bronchi, bronchiole (arrowhead), and alveoli (star) in gentamicin control negative group (G7). H&E, scale bar 100 μm.

In *K. pneumoniae* infection G1, histopathological examination of the lung section revealed pneumonic changes that alternated with emphysematous changes. The pneumonic changes are represented by the presence of inflammatory exudates within alveoli, necrotic parts of the epithelial lining of alveoli, and dilated blood vessels (**Figures 4A, B**). While GEN-treated G2 restored normal pulmonary tissue architectures, some of the sections examined revealed perivascular emphysema with inflammatory cell infiltrations (**Figure 4C** **)**. In ursolic acid methyl ester-treated G3, however, normal bronchi and alveolar epithelium were observed, along with some dilated vasculatures (**Figure 4D**). The non-infected control groups (G4, G5, G6, and G7) showed normal pulmonary tissue, bronchioles, alveolar structures, alveolar ducts, and alveoli with prominent lymphoid follicles within the wall of the bronchioles (**Figures 4E–H**).

## Discussion

The search for new, effective antibiotics against MDR pathogens is the most vital issue for the treatment of infectious diseases. There has been increasing interest among researchers in screening novel bioactive secondary metabolites from *Streptomyces* species to overcome MDR pathogens (Sharma et al., 2021). Therefore, we aimed to test the antimicrobial activity of metabolite extracts of antibiotic-producing Actinobacteria identified as *S*. *misakiensis* and *S*. *coeruleorubidus* that were isolated from soil samples in Egypt. The identification of the most active *Streptomyces* species isolates was done based on their morphological and physiological characteristics and molecular methods. The best media were ISP-1, ISP-5, and ISP-6, as they contain glucose as a simple carbon source and malt and yeast extract as organic nitrogen sources, which can increase the ability of Actinobacteria growth and pigment production, as well as the production of antibacterial agents. These findings are consistent with those obtained by Manikkam et al. (2014) and Charousová et al. (2017).

*S*. *misakiensis* exhibited strong antibacterial and antifungal activities (**Table 1S**), implying the production of various broad-spectrum antimicrobial secondary metabolites inhibiting *S. aureus*, *B*. *cereus*, *L*. *monocytogenes*, *S*. *equi*, *S*. *pyogenes*, and *S. agalactiae*, *E. coli*, *F*. *columnare*, *Salmonella* species, *A*. *hydrophila*, *K*. *pneumoniae*, *P. aeruginosa*, *A*. *fumigatu*s, *A*. *niger*, *C*. *neoformans*, *C. gattii*, and *C*. *albicans*. Similarly, Charousová et al. (2017) reported that *S*. *misakiensis* isolated from the soils of coastal islands could be used as an antibiotic and enzyme producer. Nevertheless, they found that it showed high activity against Gram-positive bacteria, moderate activity against Gram-negative bacteria, yeasts, and moulds and low activity against *P. aeruginosa* and *E. coli*. The purified active metabolites were identified as ursolic acid methyl ester and tetradecamethylcycloheptasiloxane by spectroscopy analyses (**Figures S4**, **S5**). Ursolic acid methyl ester exhibited antibacterial activity against a variety of species of MDR Gram-positive and Gram-negative bacteria, with MIC values ranging from 0.125 to 2 μg/ml (**Table 1** and **Table 1S**). A naturally occurring pentacyclic triterpenoid called ursolic acid is found in many therapeutic plants. Due to its diverse properties, including anti-oxidant and anti-inflammatory, antibacterial, and antiviral properties, it has gained a lot of attention recently (Tohmé et al., 2019; Peng et al., 2021). Peng et al. (2021) found that ursolic acid maintained intestinal homeostasis and downregulated the antibiotic resistance genes in gut microflora.

Tetradecamethylcycloheptasiloxane is an oxygenated monoterpene phytocompound present in the extracts of *Terminalia arjuna* (the MIC for C*. albicans* was 25 mg/ml) and *Mesembryanthemum edule* essential oil (MIC range of 0.02-0.31 mg/ml against *Candida* species and *C. neoformans*) (Omoruyi et al., 2014; Gupta and Kumar, 2017). In contrast to these findings, lower MICs of tetradecamethylcycloheptasiloxane, identified in *S*. *misakiensis* metabolites, ranged from 0.125 to 1 μg/ml against *C. albicans*, *C. neoformans*, *C. gattii*, *A. flavus*, *A. fumigatus*, and *A. niger* (**Table 1**, **Table 1S**).

Thiocarbamic acid, *N,N*-dimethyl, S-1,3-diphenyl-2-butenyl ester is the identified bioactive metabolite of *S*. *coeruleorubidus* (**Figures S10**–**S13**). It showed antimicrobial activity against *E. coli*, *K. pneumoniae*, *A. hydrophila*, and *S. pyogenes* (MIC range 0.5 - 4 μg/ml). Some strains of *Salmonella enterica* serovars and *L*. *monocytogens* were inhibited with thiocarbamic acid, *N,N*-dimethyl, S-1,3-diphenyl-2-butenyl ester (MIC values of 1-8 μg/ml and 4-8 μg/ml, respectively). However, *S. aureus*, *S. equi*, *P. aeruginosa*, and *Cryptococcus* species were resistant to thiocarbamic acid, *N,N*-dimethyl, S-1,3-diphenyl-2-butenyl ester. Moreover, MICs ranged from 1 to 16 μg/ml for 9/19 C. *albicans* strains, 5/7 *A. niger*, and 1/3 of the tested *A. flavus* strains. Kumar and Terli (2013) discovered that the bioactive metabolite of Indian *S*. *coeruleorubidus* isolate N-ethyl-2-(2-(3-hydroxybutyl) phenoxy) acetamide has activity for *E. coli* and *B. cereus* (10 μg/ml) than for *S. aureus* (28 μg/ml), but higher MIC for *A. flavus* (35 μg/ml) and *C. albicans* (86 μg/ml), indicating that our strain has broad-spectrum antimicrobial activity. One limitation of this study has been the limited numbers of certain species in MIC testing.

Ursolic acid methyl ester exhibits the highest antibacterial activity against the tested MDR pathogens *in vitro*. Therefore, it would be effective for the treatment of community-acquired infections in septicemia caused by *S. aureus* (ursolic acid methyl ester MIC is 1 μg/ml) and pneumonia (*K. pneumoniae* MIC is 0.5 μg/ml) models in mice. In the *S. aureus* model, the colony counts from the spleen and liver of the non-treated control group (G1) were 9.75 log CFU/g and 9.65 log CFU/g, respectively, which indicated septicemia. These results are consistent with Pollitt et al. (2018) observations of increased colony count in the liver and spleen of mice in the *S. aureus* septicemia model (6×10^5^ - 2×10^5^ CFU/g). While the group treated with ursolic acid methyl ester (G3) (5.67, 5.75 CFU/g) and the group treated with GEN (G2) (7.54, 7.76 CFU/g) showed rapid decreases in the bacterial burden and increases in the survival rate. However, the bacterial burden in oxacillin-treated (32 µg/ml) animals was significantly reduced (McVicker et al., 2014), and such a decrease had no effect on the disease state or mortality of the infected host.

In the control group (G1), *S. aureus* invaded the bloodstream and caused elevated liver enzymes and low protein levels (**Table 3S**), which are typically elevated in acute hepatotoxicity (Obi et al., 2004). The ursolic acid methyl ester-treated group (G3) has normal liver function, whereas the group that received GEN (G2) showed an increase in enzyme levels and a decrease in protein levels (**Table 3S**) due to the effect of GEN on hepatic cells (Huang and Schacht, 1990).

The kidney function parameters revealed elevated levels of creatinine and urea in the infected, non-treated group (**Table 4S**), possibly due to focal invasion during staphylococcal septicemia or toxin-mediated mechanisms, as in the staphylococcal toxic shock syndrome (Wippermann et al., 1991). On the other hand, the ursolic acid methyl ester-treated group (G3) has normal creatinine and urea levels. However, the GEN-treated group (G2) showed a slightly increased level of creatinine and urea as a result of accumulation in proximal tubular cells and interaction with cell membranes and organelles (Randjelovi et al., 2017).

With regard to histopathological changes in the liver of mice in the control-positive G1, multiple areas of coagulative necrosis surrounded by substantial inflammatory cell infiltrates and focal of microabscesses composed predominantly of neutrophils were observed (**Figures 2A, B**). The majority of liver cells had degenerative changes such as hydropic degenerations and steatosis. This finding could be due to the critical role of neutrophils in the defence against staphylococcal infection (Thwaites et al., 2011). According to Grosz et al. (2014), after I.P. injection of *S. aureus*, most of the bacteria were distributed in hepatic and splenic tissues, causing liver abscesses. However, both the GEN control negative (G7) and GEN- treated (G2) groups had minor changes in liver tissue (**Figures 2C, H**). This indicates an important complication of GEN.

The ursolic acid methyl ester-treated group (G3) appears slightly normal, with no signs of apparent toxicity such as inactivity and weight loss, and no apparent morphological or histopathological changes in the organs compared to the untreated control positive group (G1). Similarly, Tohmé et al. (2019) declared that ursolic acid had little or no toxicity to cells *in vitro*. The *in vivo* testing with no dose ranges for ursolic acid methyl ester and the preliminary toxicity testing is another limitation of this study.

In the *K*. *pneumoniae* model, a 2.20 and 2.02 log CFU/g significant reduction in CFUs, respectively, was observed in the lungs of both ursolic acid methyl ester- and GEN-treated mice compared with the untreated group (G1) (**Figure 1B**). This result is in harmony with Rodgers et al. (2019), who reported a 3.4-log reduction in lung CFU in mice treated with dissolving microarray patches containing GEN compared with their untreated counterparts.

*K. pneumoniae* has an influence on kidney function parameters in the infected, untreated group (G1) because it causes renal damage through the deposition of antigens in the kidney or the formation of immune complexes in the blood that are then accumulated in the kidney (Randjelovi et al., 2017). The ursolic acid methyl ester- treated group (G3) has normal renal and hepatic functioning. Loquat leaf extract, which is rich in ursolic acid, has been found to effectively alleviate inflammatory diseases due to inhibition of phospdiesterase-4D (Tan et al., 2017). On the other hand, the GEN-treated group (G2) had increased urea, creatinine, liver enzymes, and a lower protein level (**Table 4S**), as reported previously (Obi et al., 2004).

Extensive polymorph nuclear infiltration with pleuritis, vasculitis, and edema were observed in infected, non-treated groups (G1). It can be explained by the fact that lung epithelial cells play a significant part in the host defence mechanism against *K. pneumoniae*, using two strategies: (i) ingesting and controlling the bacteria and (ii) opsonizing the pathogen. Both methods are avoided by the capsular polysaccharide of *K. pneumoniae*, which also increases bacterial pathogenicity (Vading et al., 2018). While the GEN-and ursolic acid methyl ester-treated groups showed normal lung tissue (**Figures 4C, D**) and a high survival rate. This outcome is in line with the observations of Wang et al. (2021) that whereas GEN-treated and phage-treated animals displayed minor lung congestion at 72 h post-*K. pneumoniae* infection, the texture remained firm and glossy. There were many similarities between the histological alterations in the two treatment groups. Both revealed local capillary dilatation and a slight collapse in the local alveolar walls, but the majority of the alveolar structures remained had their normal morphology.

## Conclusion

*S. misakiensis* metabolite extracts (ursolic acid methyl ester and tetradecamethylcycloheptasiloxane) are broad*-*spectrum antimicrobial biomaterials that can be further investigated for the potential against infections caused by MDR Gram-negative and Gram-positive bacteria, yeasts, and filamentous fungi. Hence, it opens up new horizons for exploring alternative antimicrobial drugs for current and reemerging diseases.

## Data availability statement

The datasets presented in this study can be found in online repositories. The names of the repository/repositories and accession number(s) can be found in the article/**Supplementary Material**.

## Ethics statement

The animal study was reviewed and approved by Institutional Animal Care and Use Committee at Ain-Shams University (approval number ASU-SCI/MICR/2023/1/4).

## Author contributions

Conceptualization, RA, YT, ABB, G EL-D, MG, AB and HR. Methodology, RA, YT. Validation, YT, RA, ABB, G EL-D, MG, AB and HR. DNA sequence analysis, YT. Data curation, YT, RA, ABB, G EL-D, MG, AB and HR. Writing—original draft preparation, YT and RA. Writing—review and editing, YT. All authors contributed to the article and approved the submitted version.

## Supplementary material

Supplementary Figure 1 Microscopical examination of *Streptomyces* species by using light microscope and scanning electron microscope (SEM). *Streptomyces misakiensis* showing short and straight spores chains **(A)** and smooth spores in SEM analysis **(B)**. *Streptomyces coeruleorubidus* showing short and spiral spores with hook end **(C)** and spiny spores in SEM analysis **(D)**.

Supplementary Figure 2 Agarose gel electrophoresis for the amplified products of 16S rDNA gene of *Streptomyces* species. Lane M: molecular size marker, lane Pos.: positive control, lanes 1 and 2: *Streptomyces* species isolates at 1485 bp, lane Neg.: negative control.

Supplementary Figure 3 Structure of ursolic acid methyl ester and tetradecamethylcycloheptasiloxane characterized by using HPLC-preparative and G-C mass.

Supplementary Figure 4 Structure of ursolic acid methyl ester identified by using G-C mass.

Supplementary Figure 5 Structure of tetradecamethylcycloheptasiloxane identified by using G-C mass.

Supplementary Figure 6 Structures of the derivatives of ursolic acid methyl ester from S. *misakiensis* strain by IR spectrum

Supplementary Figure 7 1H Peak analysis for ursolic acid by NMR spectrum of antibiotic using chloroform as a solvent.

Supplementary Figure 8 Structures of the derivatives of tetradecamethylcycloheptasiloxane from S. *misakiensis* strain by IR spectrum.

Supplementary Figure 9 1H Peak analysis for tetradecamethylcycloheptasiloxane by NMR spectrum of antibiotic using chloroform as a solvent.

Supplementary Figure 10 Structure of thiocarbamic acid, *N,N*-dimethyl, S-1,3-diphenyl-2-butenyl ester characterized by using HPLC-preparative and G-c mass.

Supplementary Figure 11 Structure of thiocarbamic acid, *N,N*-dimethyl, S-1,3-diphenyl-2-butenyl ester identified by using G-c mass.

Supplementary Figure 12 Structures of the derivatives of thiocarbamic acid, *N,N*-dimethyl, S-1,3-diphenyl-2-butenyl ester isolated from S. *coeruleorubidus* strain using IR spectrum.

Supplementary Figure 13 1H Peak analysis for thiocarbamic acid, *N,N*-dimethyl, S-1,3-diphenyl-2-butenyl ester by NMR Spectrum of antibiotic using chloroform as a solvent.

Supplementary Table 1 Inhibition Zone diameters of *S*. *misakiensis* and *S. coeruleorubidus* and minimum inhibitory concentration values of their bioactive metabolites against the tested bacteria and fungi.
