# Supplementary figures and images for: Bioactive metabolites of Streptomyces misakiensis display broad-spectrum antimicrobial activity against multidrug-resistant bacteria and fungi
Source: Front Cell Infect Microbiol. 2023 Apr 24;13:1162721. doi: 10.3389/fcimb.2023.1162721 (PMC10165089; doi:10.3389/fcimb.2023.1162721)

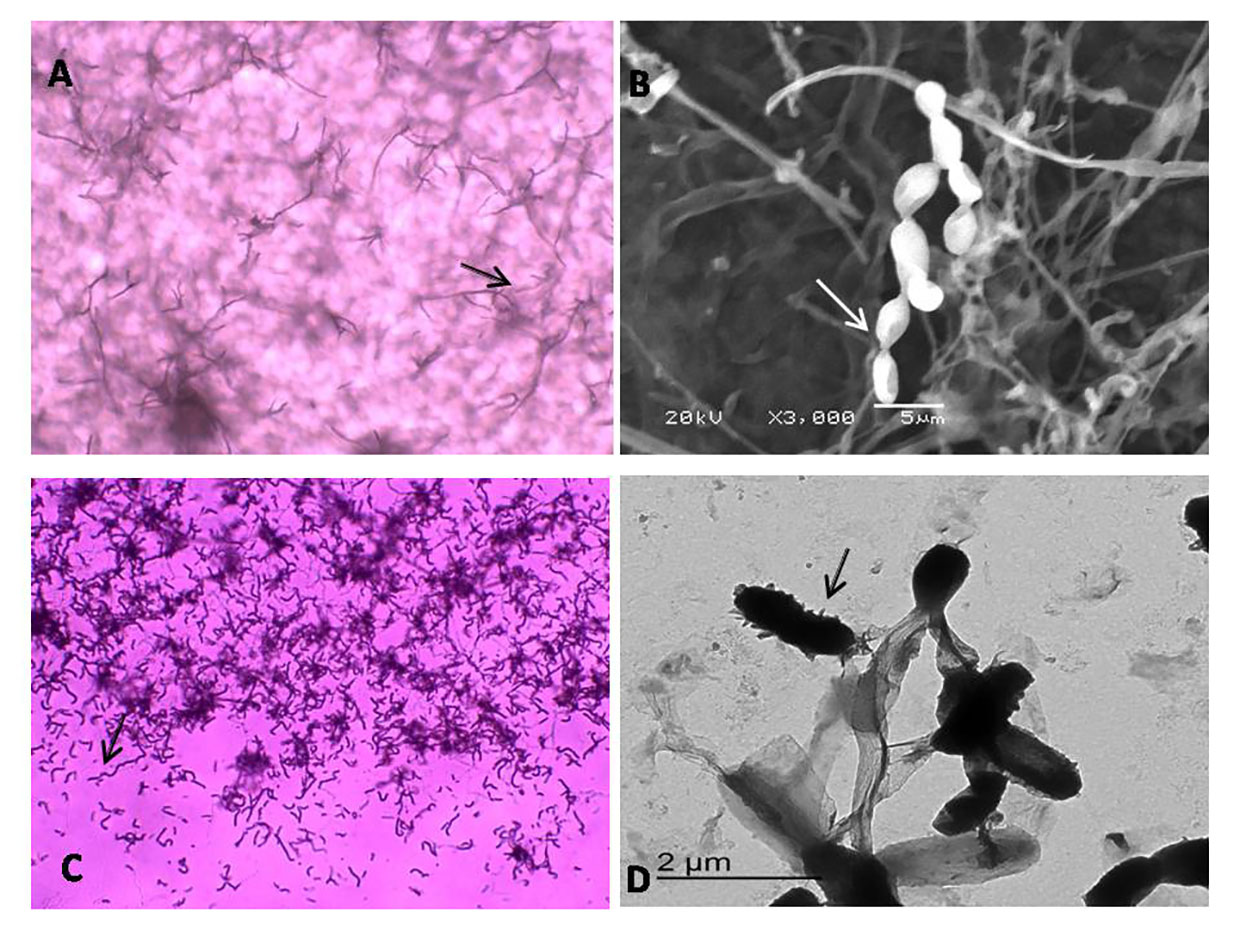

Supplement: Supplementary Figure 1 — Microscopical examination of Streptomyces species by using light microscope and scanning electron microscope (SEM). Streptomyces misakiensis showing short and straight spores chains (A) and smooth spores in SEM analysis (B). Streptomyces coeruleorubidus showing short and spiral spores with hook end (C) and spiny spores in SEM analysis (D). [file Image_1.jpeg]

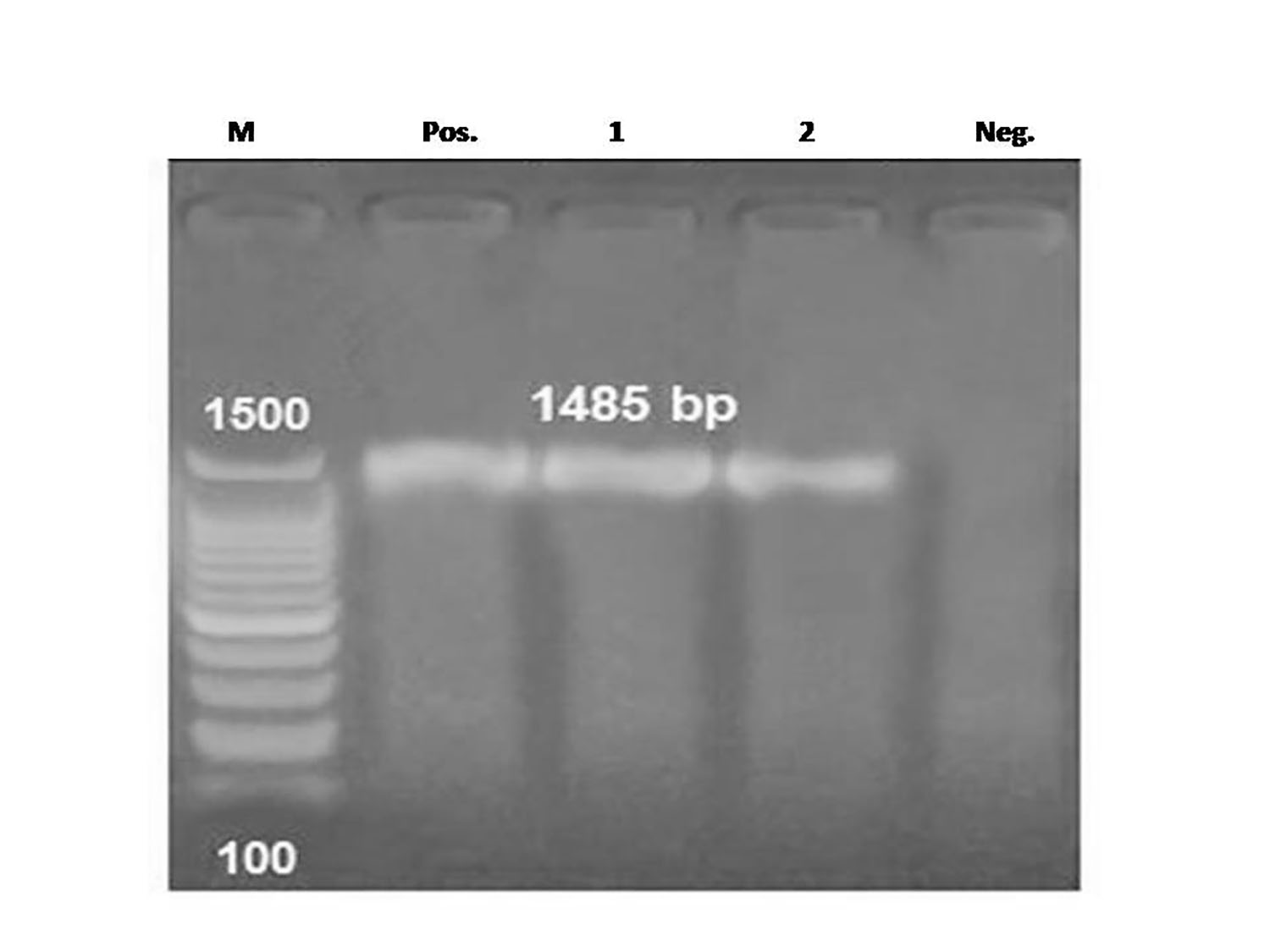

Supplement: Supplementary Figure 2 — Agarose gel electrophoresis for the amplified products of 16S rDNA gene of Streptomyces species. Lane M: molecular size marker, lane Pos.: positive control, lanes 1 and 2: Streptomyces species isolates at 1485 bp, lane Neg.: negative control. [file Image_2.jpeg]

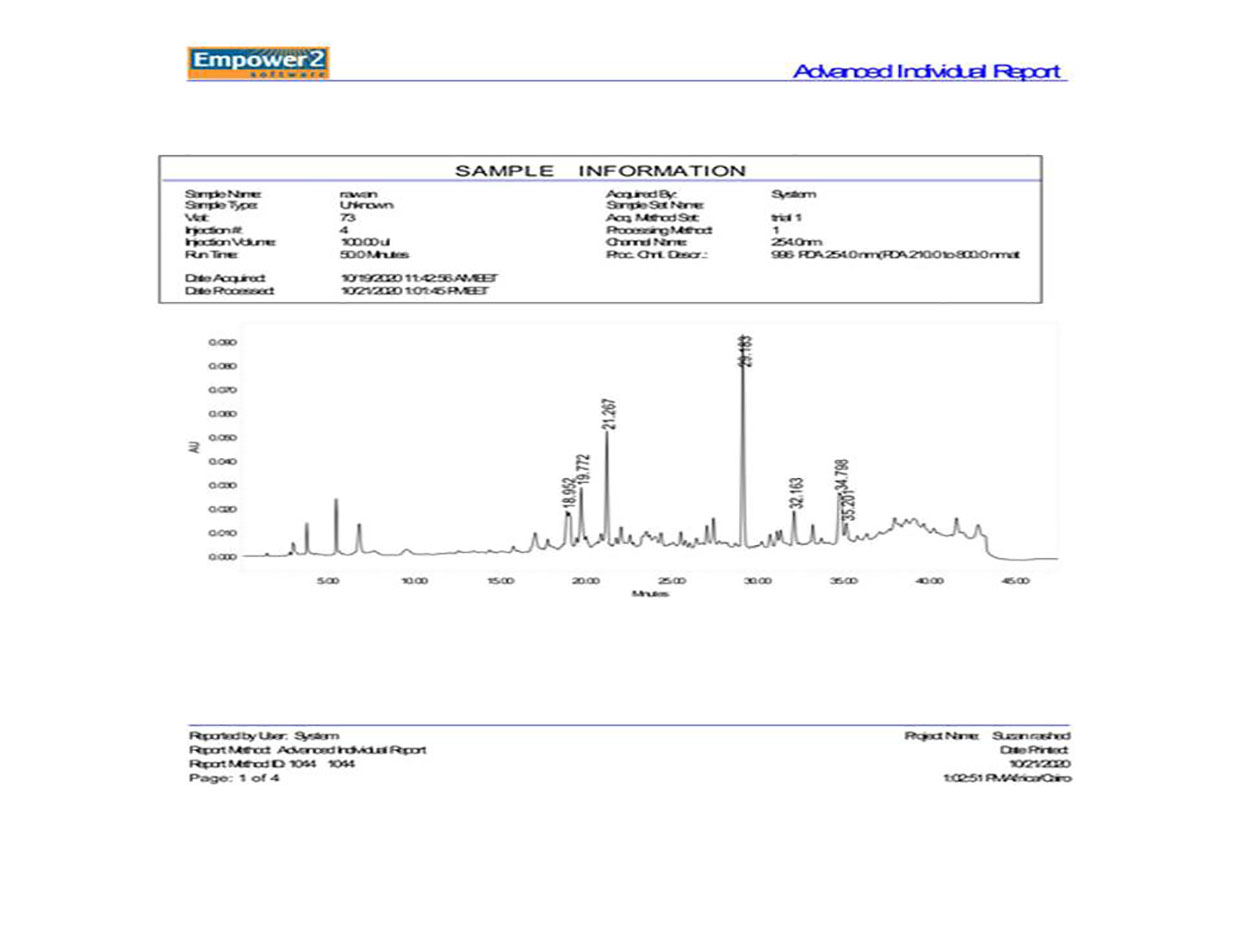

Supplement: Supplementary Figure 3 — Structure of ursolic acid methyl ester and tetradecamethylcycloheptasiloxane characterized by using HPLC-preparative and G-C mass. [file Image_3.jpeg]

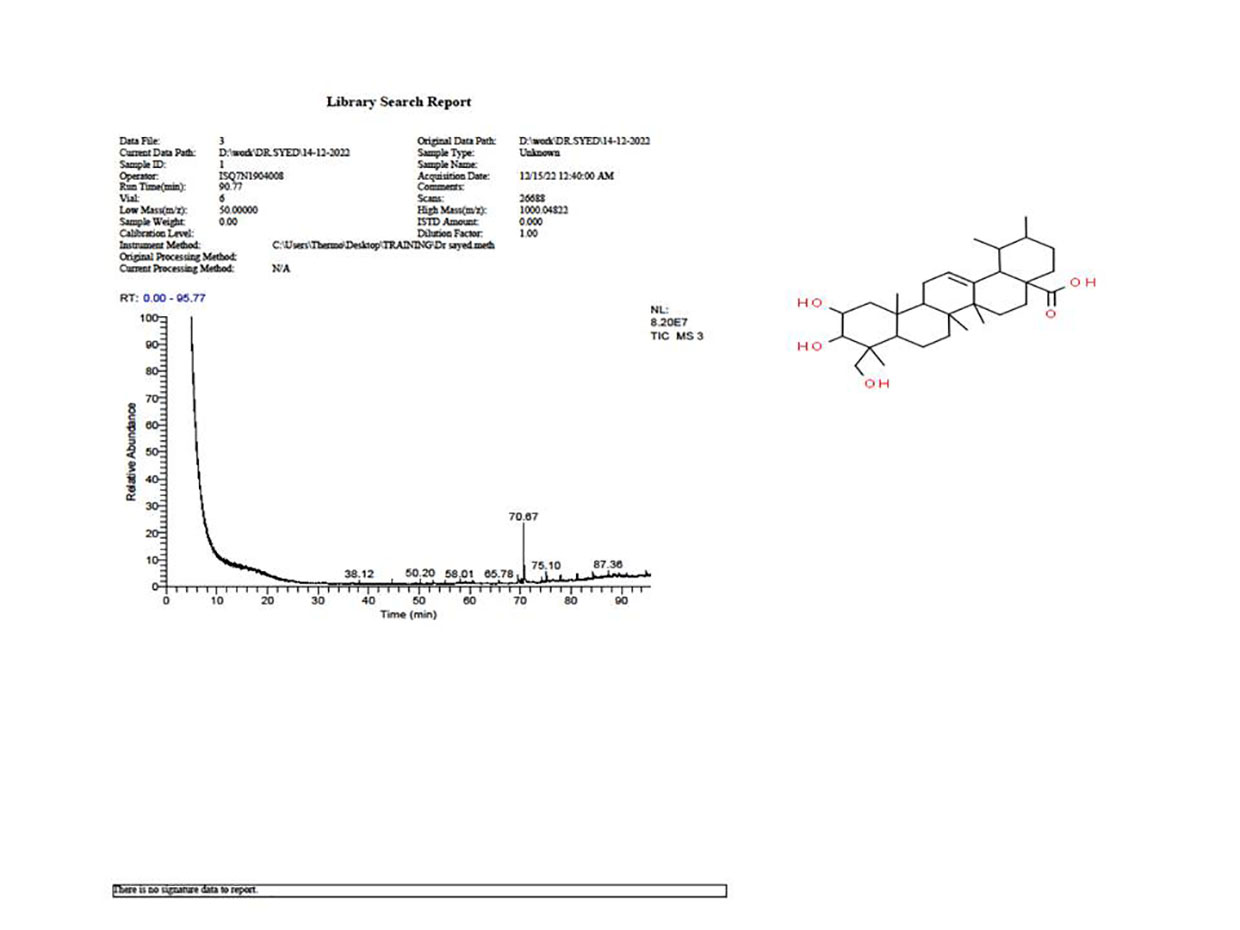

Supplement: Supplementary Figure 4 — Structure of ursolic acid methyl ester identified by using G-C mass. [file Image_4.jpeg]

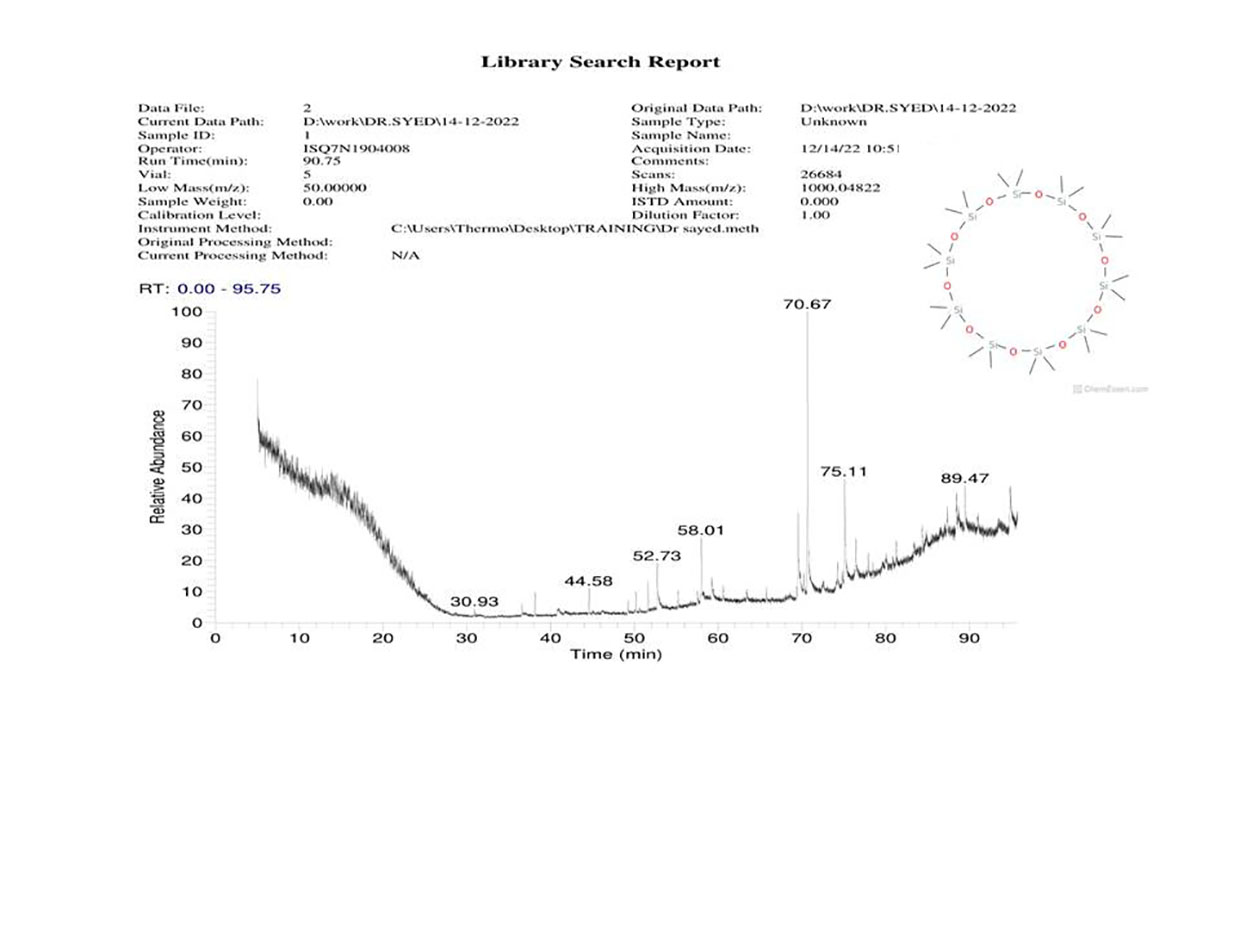

Supplement: Supplementary Figure 5 — Structure of tetradecamethylcycloheptasiloxane identified by using G-C mass. [file Image_5.jpeg]

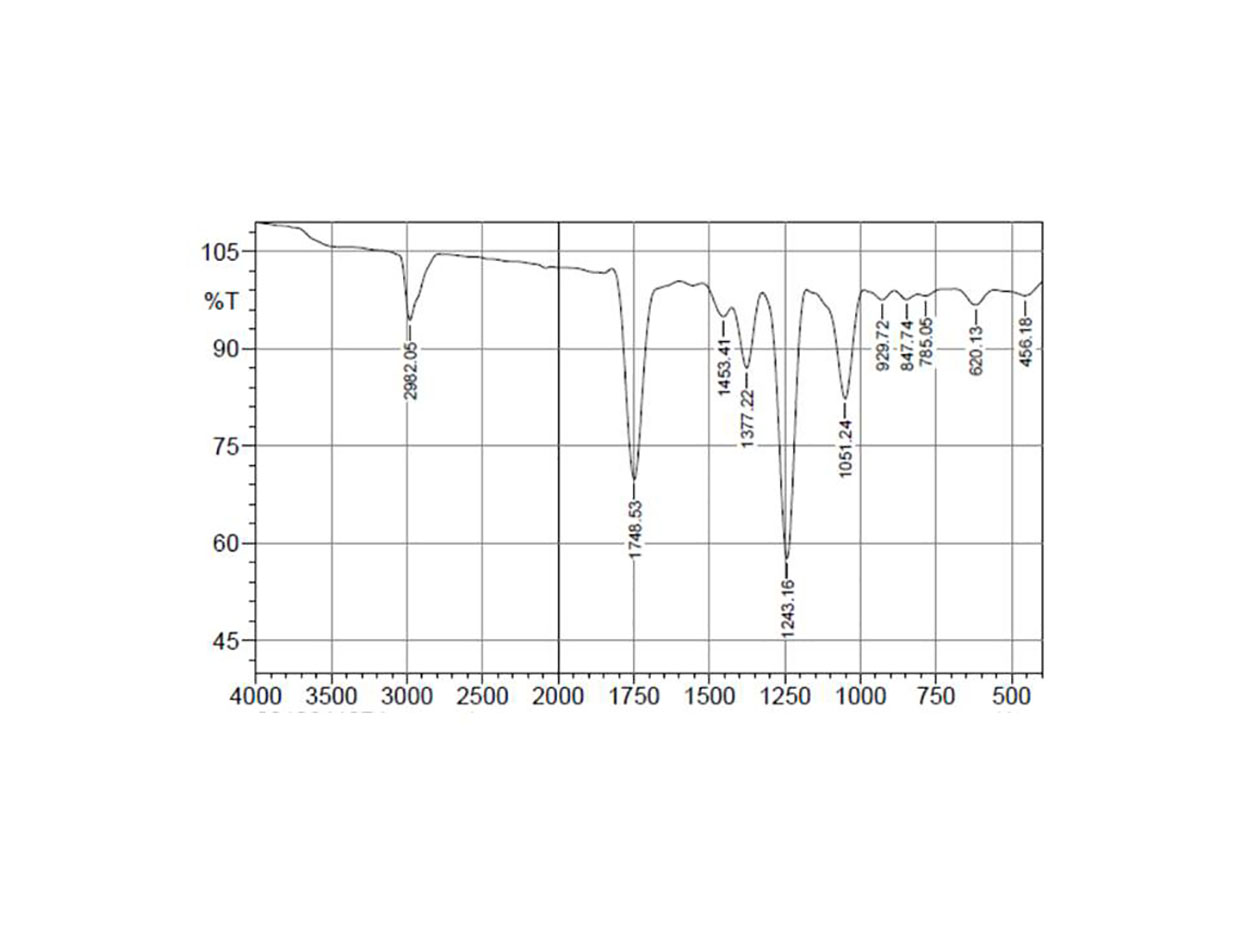

Supplement: Supplementary Figure 6 — Structures of the derivatives of ursolic acid methyl ester from S. misakiensis strain by IR spectrum [file Image_6.jpeg]

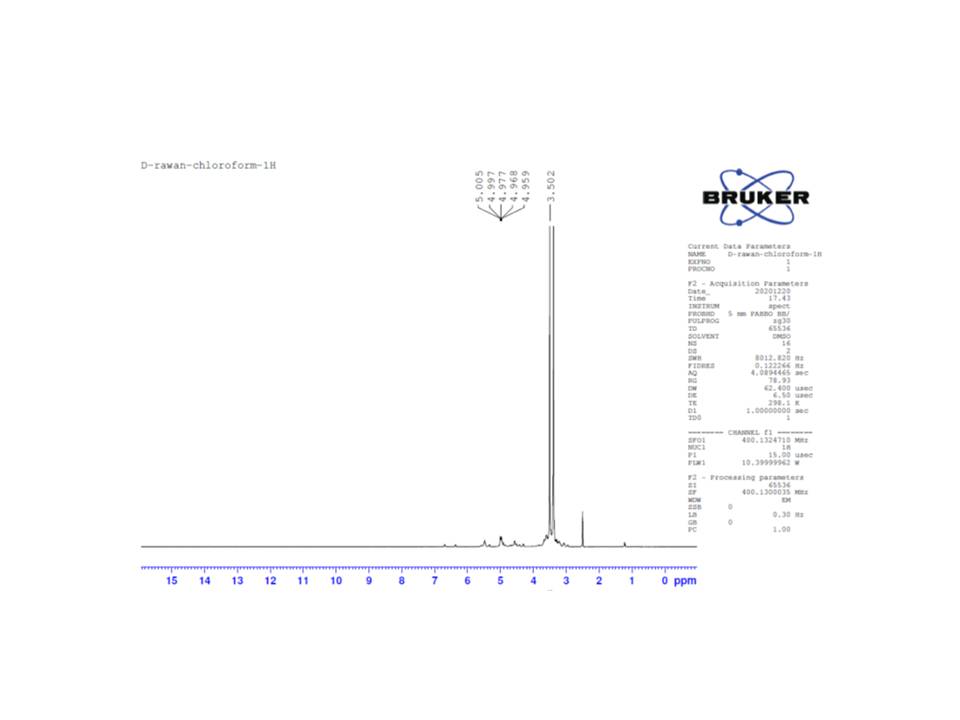

Supplement: Supplementary Figure 7 — 1H Peak analysis for ursolic acid by NMR spectrum of antibiotic using chloroform as a solvent. [file Image_7.jpeg]

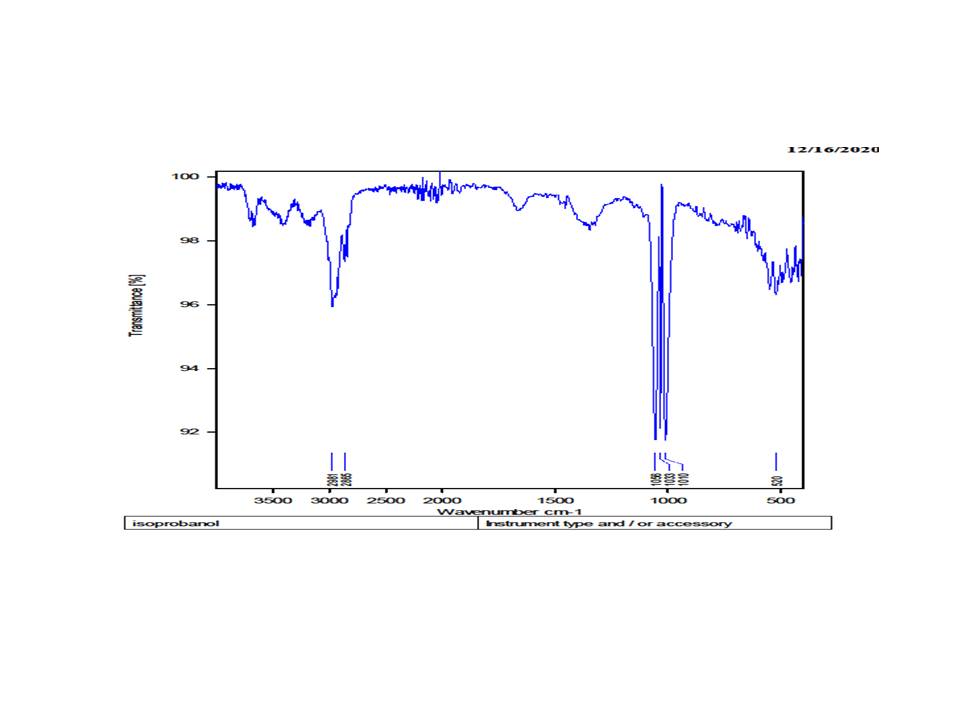

Supplement: Supplementary Figure 8 — Structures of the derivatives of tetradecamethylcycloheptasiloxane from S. misakiensis strain by IR spectrum. [file Image_8.jpeg]

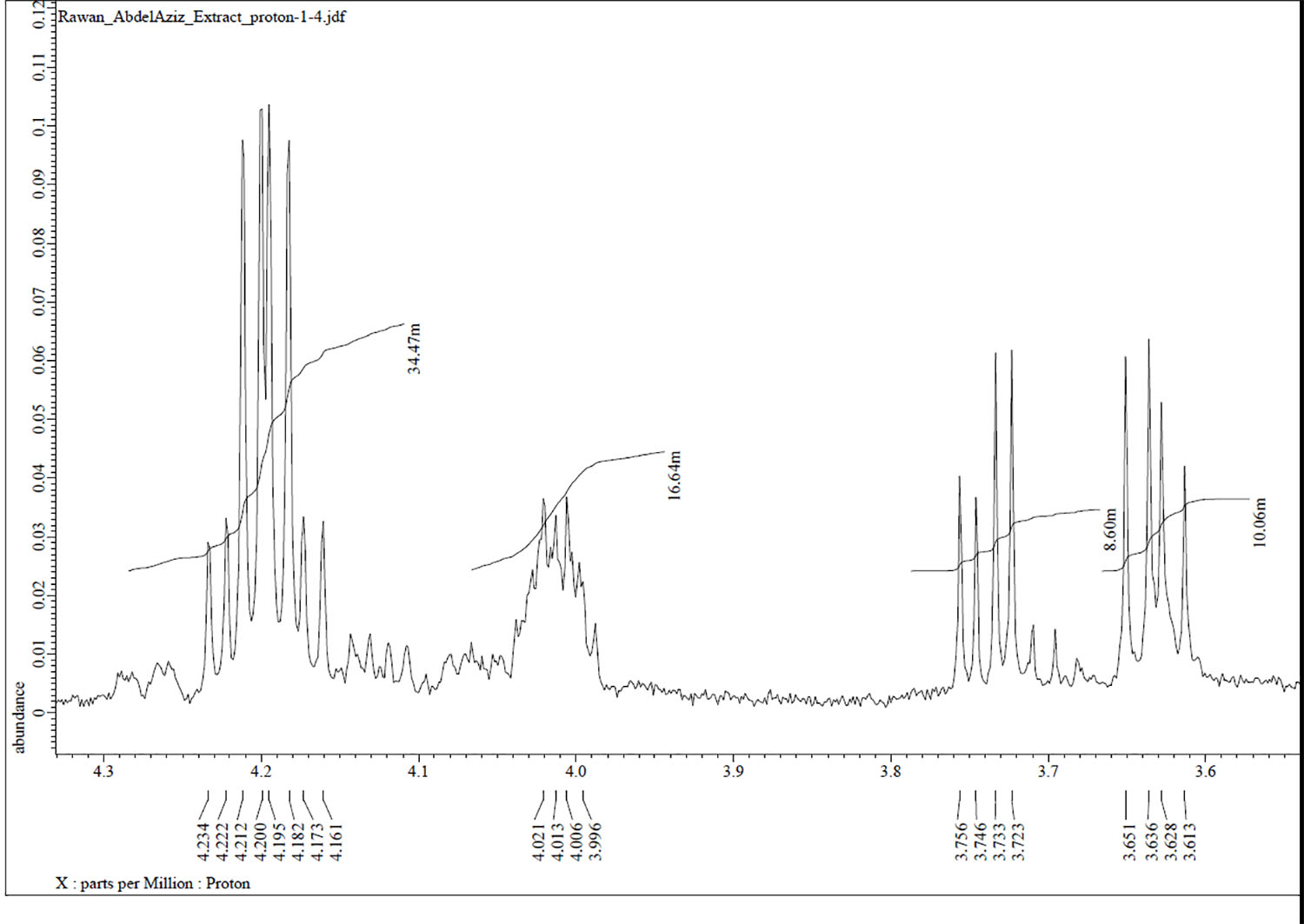

Supplement: Supplementary Figure 9 — 1H Peak analysis for tetradecamethylcycloheptasiloxane by NMR spectrum of antibiotic using chloroform as a solvent. [file Image_9.jpeg]

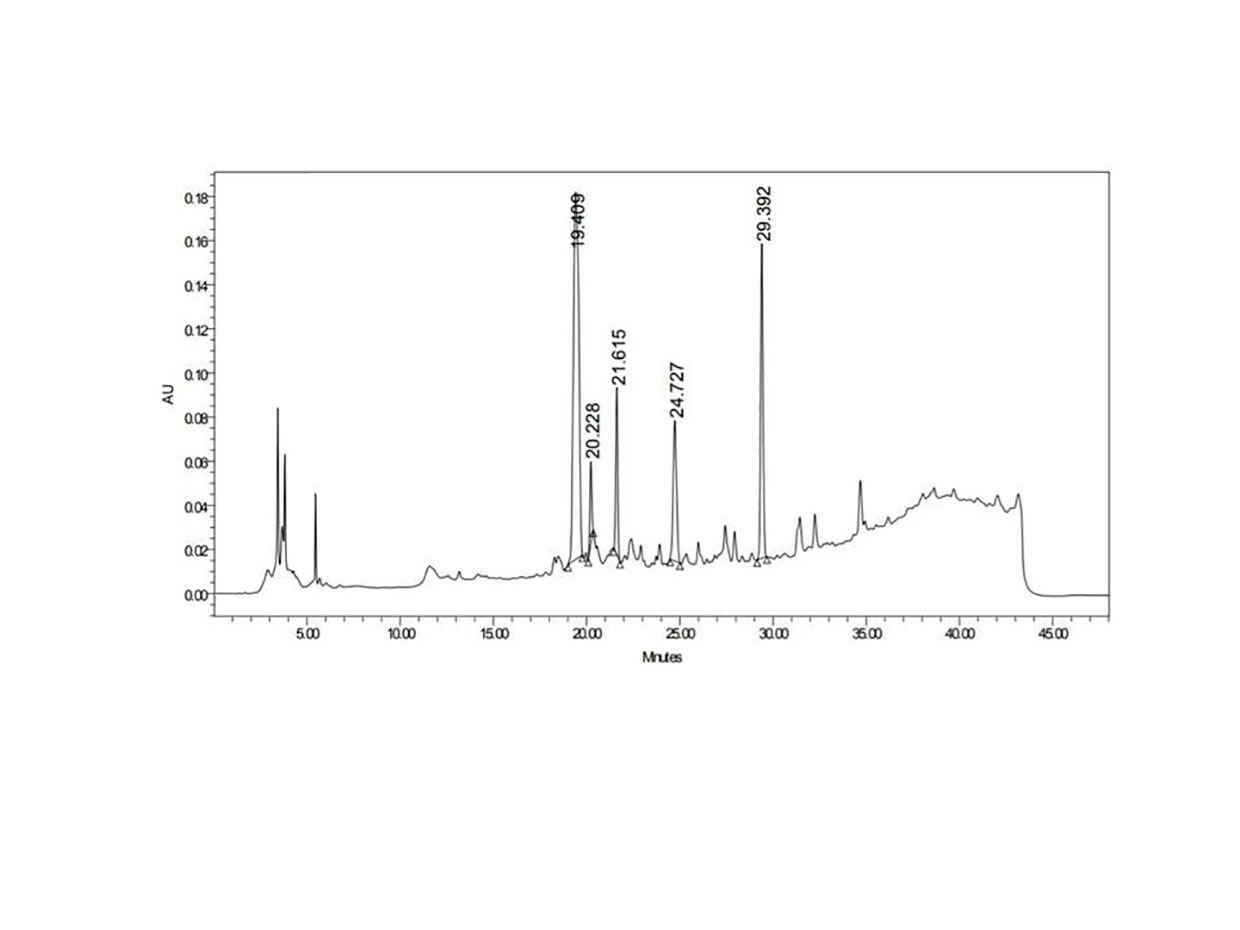

Supplement: Supplementary Figure 10 — Structure of thiocarbamic acid, N,N-dimethyl, S-1,3-diphenyl-2-butenyl ester characterized by using HPLC-preparative and G-c mass. [file Image_10.jpeg]

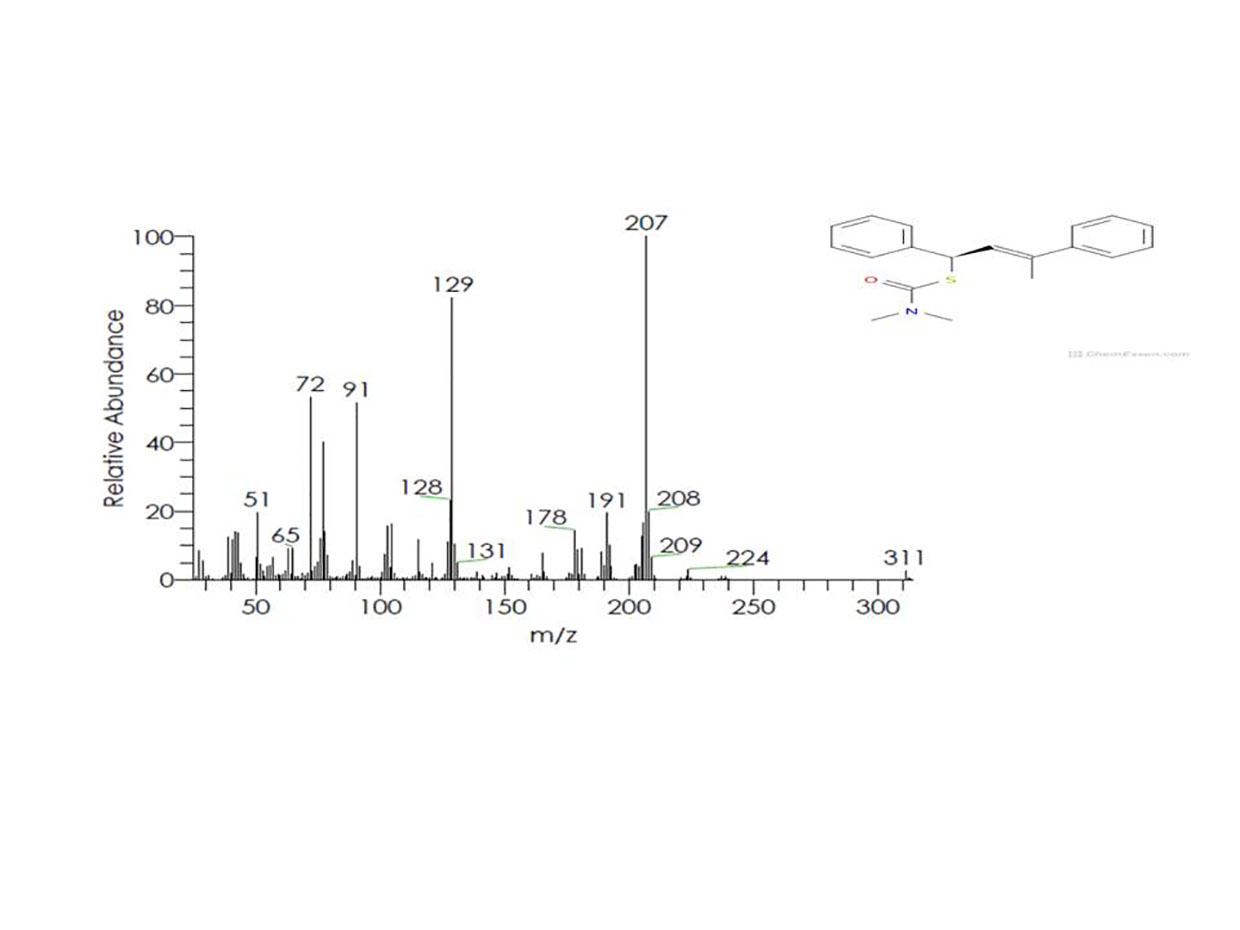

Supplement: Supplementary Figure 11 — Structure of thiocarbamic acid, N,N-dimethyl, S-1,3-diphenyl-2-butenyl ester identified by using G-c mass. [file Image_11.jpeg]

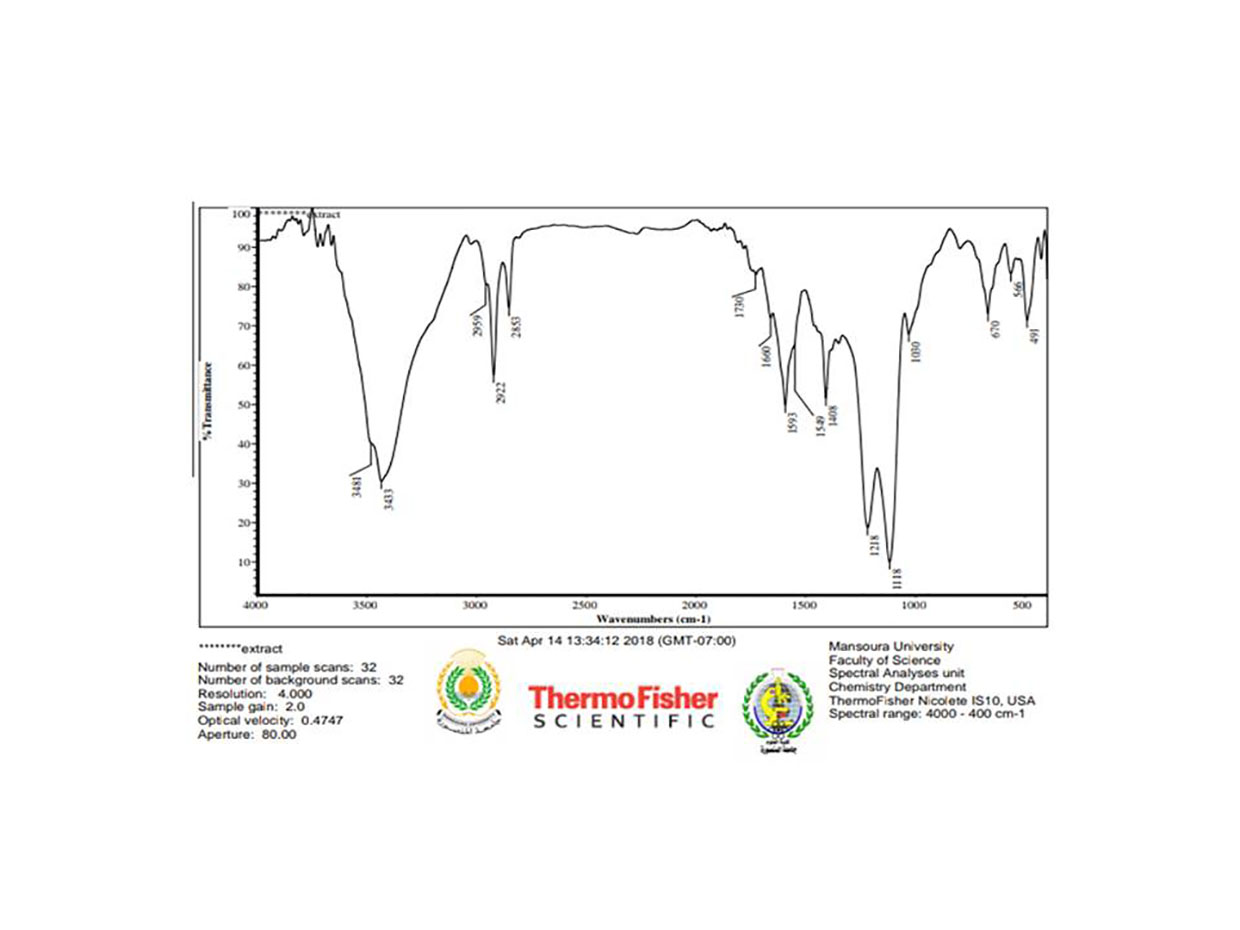

Supplement: Supplementary Figure 12 — Structures of the derivatives of thiocarbamic acid, N,N-dimethyl, S-1,3-diphenyl-2-butenyl ester isolated from S. coeruleorubidus strain using IR spectrum. [file Image_12.jpeg]

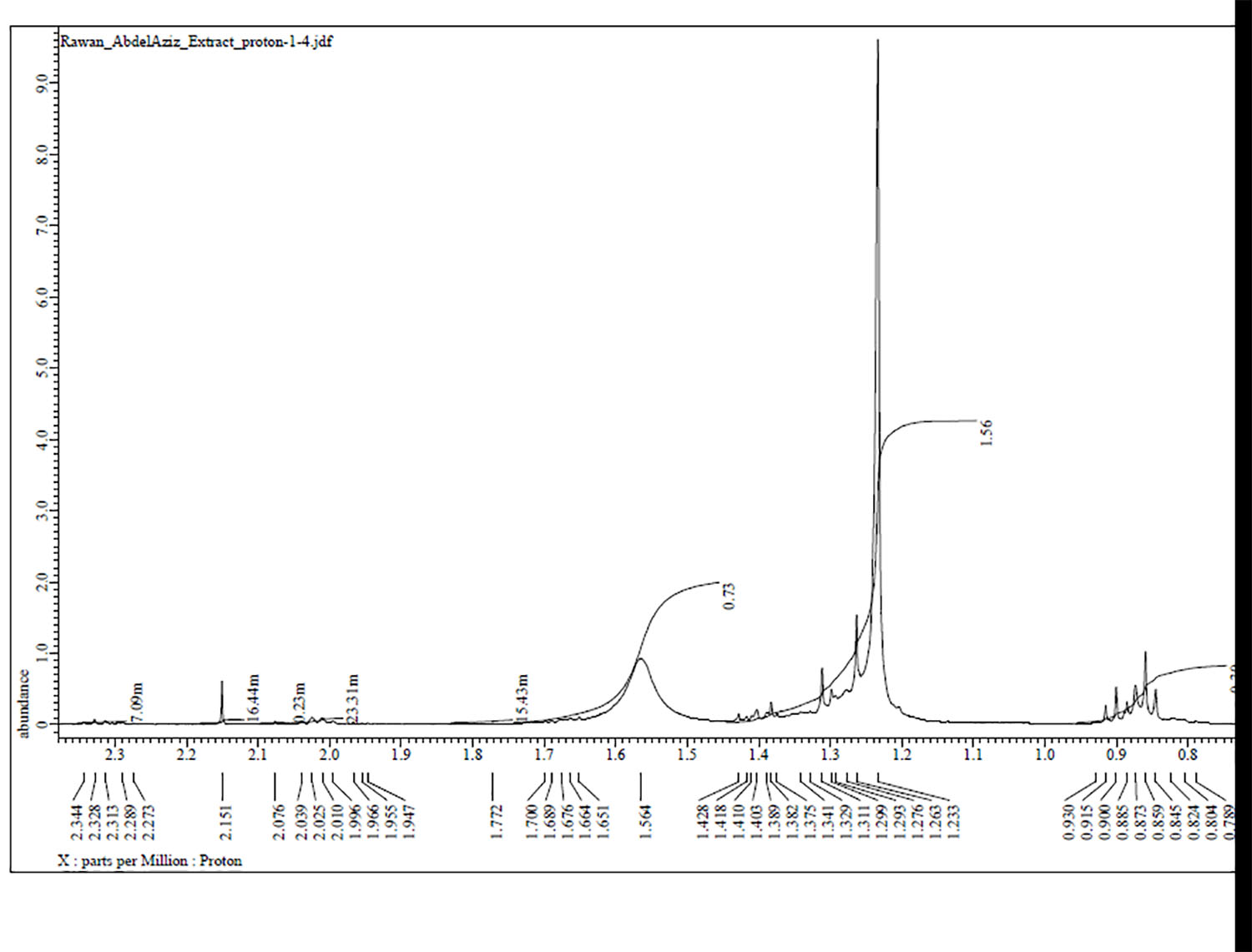

Supplement: Supplementary Figure 13 — 1H Peak analysis for thiocarbamic acid, N,N-dimethyl, S-1,3-diphenyl-2-butenyl ester by NMR Spectrum of antibiotic using chloroform as a solvent. [file Image_13.jpeg]
