# Supplementary material for: Bioactive metabolites of Streptomyces misakiensis display broad-spectrum antimicrobial activity against multidrug-resistant bacteria and fungi
Source: Front Cell Infect Microbiol. 2023 Apr 24;13:1162721. doi: 10.3389/fcimb.2023.1162721 (PMC10165089; doi:10.3389/fcimb.2023.1162721)
Supplement: Supplementary file 15 [file Table_2.doc]

**Table S2: Oligonucleotide primer sequences used in the study**

| **Target gene** | **Nucleotide sequence**  **(5′→3′)** | **Annealing temperature (°C)** | **Amplicon size (bp)** | **References** |
| --- | --- | --- | --- | --- |
| *S. aureus* *nuc* | F : GCGATTGATGGTGATACGGTT  R: AGCCAAGCCTTGACGAACTAAAGC | 55 | 447 | Brakstad *et al.,* 1992 |
| *L. monocytogenes iap* | F: ACAGCTGGGATTGCGGT  R: CCCAGCCAGAGCCGTGGA | 54 | 1395 | Comi *et al*., 1997 |
| *S. equi*  *SeeI*  *sod*A | F:GAAGGTCCGCCATTTTCAGGTAGTTG  R:GCATACTCTCTCTGTCACCATGTCCTG  F:CAGCATTCCTGCTGACATTCGTCAGG  R:CTG ACC AGC CTTATTCAC AAC CAG CC | 57 | 520  235 | Alber *et al*., 2004 |
| *Streptococcus sp*  *cfb* | F:TTTCACCAGCTGTATTAGAAGTA  R:GTTCCCTGAACATTATCTTTGAT | 55 | 153 | Ke *et al*., 2000 |
| *E. coli uidA* | F: TATGGAATTTCGCCGATTTT  R: TGTTTGCCTCCCTGCTGCGG | 55 | 166 | Heijnen and Medema, 2006 |
| *P. aeruginosa oprL* | F: ATGGAAATGCTGAAATTCGGC  R: CTTCTTCAGCTCGACGCGACG | 55 | 504 | Xu *et al*., 2004 |
| *K. pneumoniae 16S-23S ITS* | F: ATTTGAAGAGGTTGCAAACGAT  R: TTCACTCTGAAGTTTTCTTGTGTTC | 55 | 130 | Liu *et al*., 2008 |
| *Aeromonas hydrophila* 16S rRNA | F:GAAAGGTTGATGCCTAATACGTA  R: CGTGCTGGCAACAAAGGACAG | 50 | 625 | Gordon *et al*. 2007 |
| Salmonella *enterica*  *invA* | F: GTGAAATTATCGCCACGTTCGGGCA  R: TCATCGCACCGTCAAAGGAACC | 50 | 284 | De Clercq, 2007 |
| *Flavobacterium columnare*  16SrRNA | ColF: CAGTGGTGAAATCTGGT  ColR: GCTCCTACTTGCGTAGT | 45 | 675 | Darwish *et al*. 2004 |
| *Candida albicans*  ITS1-5.8S, ITS2 | F:TCCGTAGGTGAACCTGCGG  R:TCCTCCGCTTATTGAGC | 56 | 535 | [Mirhendi](https://www.frontiersin.org/articles/10.3389/fcimb.2022.807218/full?utm_source=dlvr.it&utm_medium=twitter#B28) *et al*., 2006 |
| *C. neoformans*  Aminotransferase  *C. gattii* Polymerase | *CNa-*70S: ATTGCGTCCACCAAGGAGCTC  *CNa-*70A: ATTGCGTCCATGTTACGTGGC  CNb-49S: ATTGCGTCCAAGGTGTTGTTG  CNb-49A:ATTGCGTCCATCCAACCGTTATC | 56 | 695  448 | Leal *et al.,* 2008 |
| *Aspergillus*sp*. β-tubulin* | Bt2a: GGTAACCAAATCGGTGCTGCTTTC  Bt2b:ACCCTCAGTGTAGTGACCCTTGGC | 60 | 531 *A. niger*  550 *A. flavus*  549 *A. fumigatus* | Nasri *et al*., 2015 |

F, forward; R, reverse; bp, base pair

**References**

Alber, J., El-Sayed, A., Lämmler, C., Hassan, A. A., Weiss, R., and Zschöck, M. (2004). Multiplex polymerase chain reaction for identification and differentiation of *Streptococcus equi* subsp. zooepidemicus and *Streptococcus equi* subsp. *equi. J Vet Med B Infect Dis Vet Public Health*. 51, 455–458.

Brakstad, O.G., Aasbakk, K., and Maeland, J.A. (1992). Detection of *Staphylococcus aureus* by Polymerase Chain Reaction Amplification of the *nuc* Gene. *J Clin Microbiol*.30, 1654-1660.

Comi, G. L., Cocolin, C. Cantoni, and Manzano, M. (1997). A RE-PCR method to distinguish *Listeria monocytogenes* serovars. *FEMS I munol. Med. Microbiol*. 18, 99–104.

Ke, D., Ménard, C., Picard, F.J., Boissinot, M., Ouellette, M., Roy, P.H., and Bergeron, M.G. (2000). Development of Conventional and Real-Time PCR Assays for the Rapid Detection of Group B Streptococci. *Clin. Chem*. 46, 324–331.

De Clercq, D., Ceustermans, A., Heyndrickx, M.J. Coosemans, J. Ryckeboer, A. (2007). A rapid monitoring assay for the detection of Salmonella spp. and Salmonella *Senftenberg* strain W775 in composts. *J Appl Microbiol*. 103, 1364-5072.

Darwish, A.M., Ismaiel, A.A., Newton, J.C., and Tang, J. (2004). Identification of *Flavobacterium* *columnare* by a species-specific polymerase chain reaction and renaming of ATCC 43622 strain to *Flavobacterium* *johnsoniae*. *Mol Cell Probes* 18, 421-427.

Gordon, L., Giraud, E., Ganière, J. P., Armand, F., Bouju-Albert, A., De La Cotte, N., et al. (2007). Antimicrobial Resistance Survey in a River Receiving Effluents from Freshwater Fish Farms. *J. Appl. Microbiol*. 102, 1167–1176.

Heijnen, L. and Medema, G. (2006). Quantitative detection of *E. coli*, *E. coli* O157 and other shiga toxin producing *E. coli* in water samples using a culture method combined with real-time PCR. *J Water Health*. 4, 487-498.

Leal, A.L., Faganello, J., Bassanesi, M.C., and Vainstein, M.H. (2008). *Cryptococcus* species identification by multiplex PCR. *Med Mycol*. 46, 377– 383.

Liu, Y., Liu, C., Zheng, W., Zhang, X., Yu, J., Gao, Q., et al. (2008). PCR Detection of *Klebsiella pneumoniae* in Infant Formula Based on 16S-23S Internal Transcribed Spacer. *Int. J. Food Microbiol.* 125, 230–235. doi:10.1016/j.ijfoodmicro.2008.03.005.

Mirhendi, H., Makimura, K., Khoramizadeh, M., and Yamaguchi H. (2006). A One-enzyme PCR-RFLP assay for identification of six medically important *Candida* species. *Jpn J Med Mycol*. 47, 225-229.

Nasri, T., Hedayat, M.T., Abastabar, M., Pasqualotto, A.C., Armak, M.T., and Nabıli, M. (2015). PCR-RFLP on β-tubulin gene for rapid identiﬁcation of the most clinically important species of Aspergillus*. J Microbiol Meth*. 117, 144–147.

Xu, J., Moore, J. E., Murphy, P. G., Millar, B. C., and Elborn, J. S. (2004). Early Detection of *Pseudomonas aeruginosa* - Comparison of Conventional versus Molecular (PCR) Detection Directly from Adult Patients with Cystic Fibrosis (CF). *Ann. Clin. Microbiol. Antimicrob.* 3, 1186-1476.
