# Supplementary material for: Bioactive metabolites of Streptomyces misakiensis display broad-spectrum antimicrobial activity against multidrug-resistant bacteria and fungi
Source: Front Cell Infect Microbiol. 2023 Apr 24;13:1162721. doi: 10.3389/fcimb.2023.1162721 (PMC10165089; doi:10.3389/fcimb.2023.1162721)
Supplement: Supplementary file 16 [file Table_3.doc]

**Table 3S:** Liver and kidney function parameters of mice infected via the intraproteinial route with *S. aureus* and treated with gentamicin (20 mg/kg) and/or ursolic acid methyl ester (10 mg/kg).

| **Groups** | **ALT** | **AST** | **Albumin** | **Total protein** | **Total bilirubin** | **Direct bilirubin** | **Urea** | **Creatinine** |
| --- | --- | --- | --- | --- | --- | --- | --- | --- |
| **G1** | 52.87±0.975^b^ | 63.33±0.698b | 2.42±0.277b | 1.31±0.075c | 1.90 ±0.254c | 0.20±0.057c | 58.50±0.952b | 1.87±0.086b |
| **G2** | 84.37±0.369a | 92.17±1.333a | 1.11±0.057c | 3.47±0.230b | 1.83±0.076c | 0.90 ±0.54ab | 61.90±0.606a | 2.30 ±0.254a |
| **G3** | 27.57±1.050c | 38.34±0.282c | 3.30±0.150a | 5.07±0.121a | 2.70±0.173b | 1.43±0.242a | 40.33±0.282c | 1.13±0.077c |
| **G4** | 17.75±0.207d | 33.00±0.987d | 3.46±0.150a | 5.51±0.115a | 1.23±0.086d | 0.70 ±0.050b | 39.63±0.259c | 0.89 ±0.103c |
| **G5** | 52.67±1.154b | 61.97±1.674b | 2.10±0.150b | 3.33±0.242b | 2.13±0.086c | 1.23±0.086a | 58.44±0.594b | 1.90±0.054b |
| **G6** | 16.28±0.381d | 16.28±0.381f | 3.33±0.051a | 3.25±0.127b | 5.50 ±0.162a | 1.15±0.28a | 39.70±0.433c | 0.99±0.053c |
| **G7** | 15.61±0.254d | 23.87±0.086e | 3.32±0.144a | 5.04±0.092a | 1.15 ±0.028d | 0.14±0.023c | 39.30±0.352c | 0.90±0.055c |

G1: Infected non-treated group; G2: Gentamicin-treated group, G3: Ursolic acid methyl ester*-*treated group; G4: Ursolic acid methyl ester-control negative group; G5: Gentamicin-control negative group; G6: Saline-control negative group; G7: Tween 20-control negative group. ^a^ Significant viability decrease with *P* < 0.05 in G2 and G3 versus G1. ^b^ Significant viability decrease with *P<* 0.05 in G3 versus G2. Values having different superscripts within the same column are significantly different (*P* < 0.05). SE: standard error.
