# Supplementary material for: Bioactive metabolites of Streptomyces misakiensis display broad-spectrum antimicrobial activity against multidrug-resistant bacteria and fungi
Source: Front Cell Infect Microbiol. 2023 Apr 24;13:1162721. doi: 10.3389/fcimb.2023.1162721 (PMC10165089; doi:10.3389/fcimb.2023.1162721)
Supplement: Supplementary file 17 [file Table_4.doc]

**Table 4S:** Liver and kidney function parameters of mice infected intranasally with *K. pneumoniae* and treated with gentamicin (10 mg/kg) and/or ursolic acid methyl ester (7.5 mg/kg)

| **Groups** | **ALT** | **AST** | **Albumin** | **Total protein** | **Total bilirubin** | **Direct bilirubin** | **Urea** | **Creatinine** |
| --- | --- | --- | --- | --- | --- | --- | --- | --- |
| **G1** | 42.49±0.606**^c^** | 58.27±0.433**^b^** | 1.33±0.121**^b^** | 2.78±0.080**^d^** | 2.40±0.057**^a^** | 1.13±0.184**^a^** | 56.63 ±0.502ab | 1.73±0.069b |
| **G2** | 57.43±1.131**^a^** | 65.23±1.068**^a^** | 3.42±0.057**^a^** | 2.70±0.254**^d^** | 1.38±0.046**^b^** | 0.57 ±0.282**^bc^** | 58.13±1.062a | 1.93±0.069a |
| **G3** | 27.57±1.050^d^ | 38.60±0.848**^d^** | 3.40±0.063**^a^** | 3.60±0.103**^c^** | 1.23±0.34**^cd^** | 0.85 ±0.028**^ab^** | 38.87±0.588c | 0.80±0.057c |
| **G4** | 16.51±1.091^e^ | 37.95±0.329^d^ | 3.38±0.057^a^ | 5.78±0.109^a^ | 1.03±0.034^e^ | 0.33±0.086^c^ | 36.47±1.141c | 0.77±0.034c |
| **G5** | 46.33±0.508**^b^** | 54.27±0.560**^c^** | 3.37±0.017**^a^** | 5.74±0.132**^a^** | 1.30±0.053**^dc^** | 0.89 ±0.046**^ab^** | 54.67±0.646b | 1.93±0.034a |
| **G6** | 16.28±0.381**^e^** | 37.23±0.352**^d^** | 3.25±0.127**^a^** | 5.7 ±0.034**^a^** | 1.10±0.052**^d^** | 0.38 ±0.161**^bc^** | 39.33±0.600c | 0.93 ±0.086c |
| **G7** | 15.61±0.254**^e^** | 23.87±0.086**^e^** | 3.32±0.144**^a^** | 5.21±0.005**^b^** | 1.15±0.028**^d^** | 0.14 ±0.023**^c^** | 39.30±0.352c | 0.90±0.057c |

G1: Infected non-treated group; G2: Gentamicin-treated group, G3: Ursolic acid methyl ester*-*treated group; G4: Ursolic acid methyl ester-control negative group; G5: Gentamicin-control negative group; G6: Saline-control negative group; G7: Tween 20-control negative group. ^a^ Significant viability decrease with *P* <0.05 in G2 and G3 versus G1. ^b^ Significant viability decrease with *P<* 0.05 in G3 versus G2. Values having different superscripts within the same column are significantly different (*P* < 0.05). SE: standard error.
